# Personalized *in silico* modeling of cardiac ion channel variants to predict drug-induced proarrhythmia risk

**DOI:** 10.3389/fphys.2026.1828506

**Published:** 2026-08-04

**Authors:** Alia Henedi, Jalal Cherkaoui, Stelian Camara Dit Pinto, Steven M. Levine, Mohammed Cherkaoui, Nicolas R. Gallo, Kenza E. Benzeroual

**Affiliations:** 1 Department of Pharmaceutical Sciences, Arnold & Marie Schwartz College of Pharmacy and Health Sciences, Long Island University, Brooklyn, NY, United States; 2 School of Engineering, Computer Science and Artificial Intelligence, Long Island University, Brooklyn, NY, United States; 3 Dassault Systemes, Waltham, MA, United States

**Keywords:** amiodarone, APD90, cardiac ion channels variants, drug-induced cardiotoxicity (DICT), genetic variations, hERG, *in silico* models, Nav1.5

## Abstract

**Introduction:**

Drug-induced cardiotoxicity remains a leading cause of drug development failures and market withdrawals. Despite advances in preclinical testing, current *in-silico* models often overlook genetic variability, limiting their ability to capture patient-specific responses. We present a computational modeling framework that integrates known genetic variants in cardiac ion channels to predict individual differences in drug-induced arrhythmogenic risk.

**Methods:**

We simulated concentration-dependent effects of amiodarone across combinations of hERG and Nav1.5 alleles in five human cardiac cell types: endocardial, epicardial, midwall, Purkinje, and atrial cells. APD90 values were evaluated across all cell types, whereas qNet was calculated in ventricular cells only to assess genotype and cell type dependent differences in electrophysiological response and torsadogenic risk.

**Results:**

Our results highlight how genetic variations and cell type context influence electrophysiological response to amiodarone, uncovering high-risk profiles otherwise masked in population-averaged models. These simulations reveal key insights with translational and regulatory relevance: genetic background meaningfully alters drug response; midmyocardial cells are disproportionately vulnerable; the same mutation can produce different effects across cell types; Purkinje cells may serve as silent proarrhythmic substrates; and celltype- specific differences in APD90 and qNet may provide additional insight beyond APD prolongation alone, and consequently improve prediction of torsades de pointes risk.

**Conclusion:**

Our work provides a foundation for the creation of digital twin models that incorporate patient-specific electrophysiology, offering a scalable platform for *in-silico* cardiac safety. By linking genetic polymorphisms to context-dependent functional outcomes, this approach supports early-stage candidate prioritization, offers a scalable platform for genotype-specific risk stratification, and advances the implementation of precision cardiotoxicity screening in drug development and clinical safety assessments.

## 1 Introduction

Drug-induced cardiotoxicity (DICT) remains a persistent barrier to drug development, accounting for a significant proportion of late-stage clinical trial failures and post-market withdrawals (Ferri et al., 2013; Laverty et al., 2011; Onakpoya et al., 2016; Mamoshina et al., 2021). Among its clinical manifestations, drug-induced torsades de pointes (TdP) poses a particular concern due to its association with sudden cardiac death (Cubeddu, 2003; Gintant, 2008; Herrmann et al., 2022; Li et al., 2022). Regulatory guidelines such as ICH E14 and S7B have historically relied on QT prolongation and hERG channel inhibition as key surrogate markers for arrhythmogenic risk. However, while sensitive, these markers suffer from limited specificity (Sager et al., 2014; Colatsky et al., 2016). As a result, potentially safe compounds may be prematurely discontinued, while others may advance despite hidden risks.

Recognizing the limitations of a singular focus on hERG blockade, the Comprehensive *in vitro* Proarrhythmia Assay (CiPA) was introduced in 2013 to modernize preclinical cardiac safety testing. CiPA integrates multichannel *in vitro* data and *in silico* modeling to improve risk stratification (Taboureau and Jorgensen, 2011; Lawrence et al., 2005; Hoffmann and Warner, 2006; Yim, 2018). Yet despite these advances, a key limitation remains unaddressed: the lack of consideration for genetic variability in cardiac ion channels across diverse populations. Existing *in silico models* typically simulate responses in a genetically uniform individual, failing to reflect the full spectrum of ion channel polymorphisms observed in clinical populations (Evelyn et al., 2001; Florian et al., 2011; Grosjean and Urien, 2012). Britton et al. showed that incorporating electrophysiological variability can improve representation of intersubject differences and variability in drug response, although genotype-specific integration of ion channel variants remains limited (Britton et al., 2013). This gap is particularly concerning given the underrepresentation of diverse populations in clinical trials and growing recognition of pharmacogenomics as a driver of inter-individual variability in drug response (Allison et al., 2022; Clark et al., 2019).

Cardiac ion channels such as Nav1.5 (encoded by *SCN5A*) and hERG/Kv11.1 (encoded by *KCNH2*) play essential roles in shaping the cardiac action potential. Mutations in these channels underlie inherited channelopathies such as LQT2 and LQT3, and they directly modulate the electrophysiological response to pharmacological blockade (Sanguinetti et al., 1996; Bezzina et al., 2003; Makielski et al., 2003; Anson et al., 2004; Kass, 2005; Ravens and Cerbai, 2008; Ruan et al., 2009). Amiodarone, a broadly used antiarrhythmic drug with known torsadogenic potential (Woosley and Romer, [[NoYear]]; Kodama et al., 1997; Florek et al., 2024), exhibits a multichannel blockade profile including hERG (IKr), Nav1.5 (INa), and Cav1.2 (ICaL). This makes it an ideal compound to examine pharmacogenetic interactions, since the blockade of inward calcium and sodium currents may partially counterbalance the proarrhythmic effects of hERG (IKr) inhibition (January and Riddle, 1989; Wu et al., 2008). Prior studies have described variability in ion current block due to single mutations, but the combined impact of multiple channel variants on cellular electrophysiology and TdP risk remains poorly understood.

**Figure 1:**
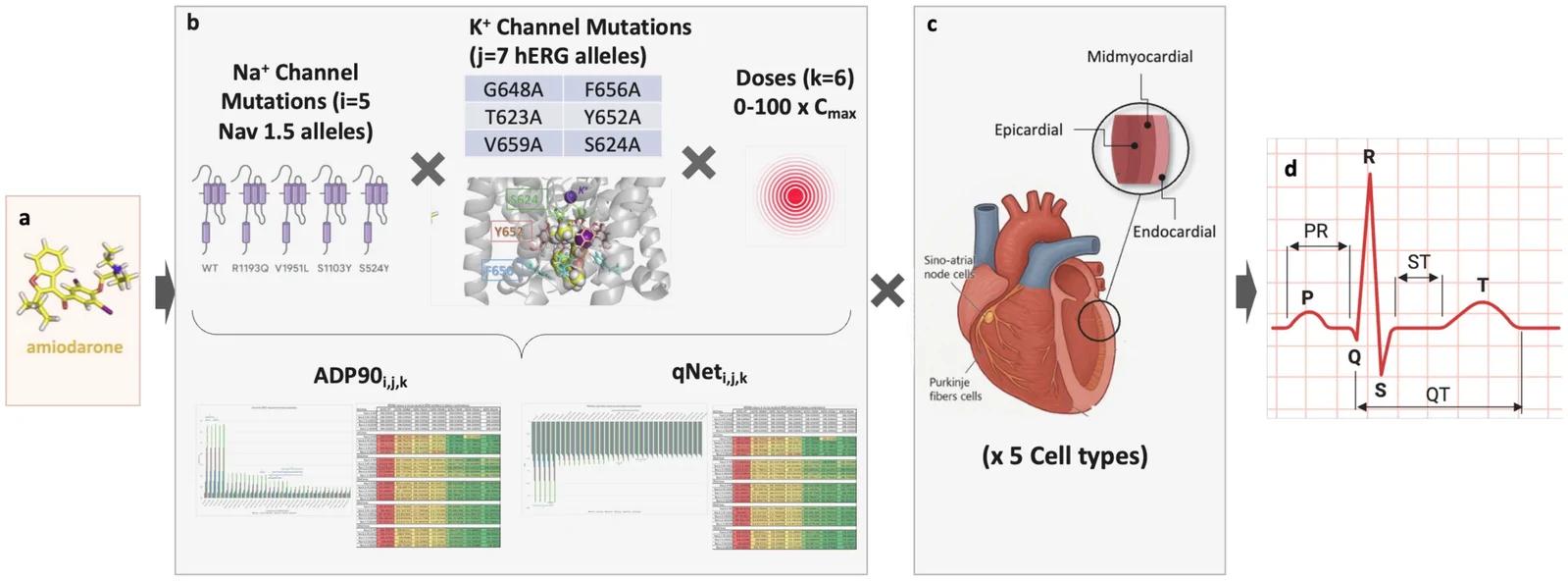
Workflow for individualized cardiotoxicity prediction by integrating genetic variation into digital heart modeling. **(a)** Candidate drug input—here, amiodarone—is selected based on its known multichannel blocking properties and clinical relevance. **(b)** For each of the six concentrations (k = 6), APD90 was simulated for all combinations of seven hERG and five Nav1.5 channel alleles (j × i permutations). qNet was simulated for the same allele combinations for ventricular cells only. Both outcomes were calculated using published IC50 values. **(c)** APD90 simulations were performed in five human cardiac cell types—endocardial, epicardial, midwall, Purkinje, and atrial—to capture cell-type-specific responses, whereas qNet was calculated only in the three ventricular cell types. **(d)** Outputs can be integrated into a whole-heart digital model to simulate the ECG in future work, enabling quantification of QT interval prolongation and inference of torsadogenic risk.

In this study, we present a multi-cell, allele-specific modeling approach to quantify the impact of genetic variability on the cardiotoxic potential of amiodarone. Using combinations of hERG and Nav1.5 alleles, we simulated action potential duration at 90% repolarization (APD90) and computed qNet (a CiPA-endorsed digital biomarker) for multiple cardiac cell types. By incorporating published IC_50_ values for variant-specific drug-channel interactions, our model provides a physiologically grounded and personalized framework to assess arrhythmia risk in silico. The goal of this work is to demonstrate the feasibility of integrating genetic information into digital heart models to enhance cardiotoxicity prediction and move toward individualized drug safety screening. Rather than providing an exhaustive catalog of mutation-drug interactions, this study illustrates how in silico methods can serve as flexible, testable platforms to model the personalized and tissue-specific variability that underlies drug-induced arrhythmia (**Figure 1**). This positions the methodology as a potential complement to current safety pipelines.

## 2 Methods

**Figure 2:**
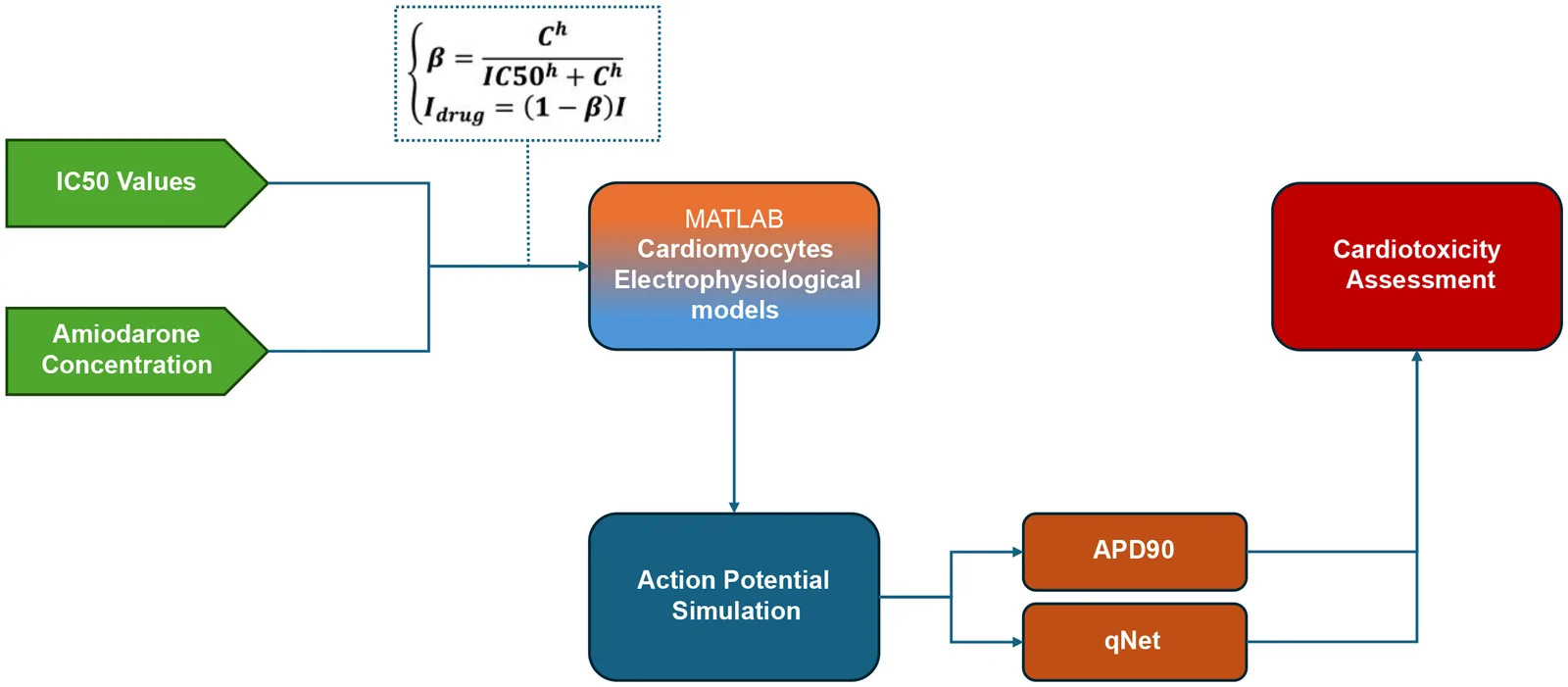
Methodology pipeline.

To evaluate the impact of drug-induced ion channel blockade on cardiac electrophysiology, we simulated the action potential (AP) of ventricular cardiomyocytes (epicardial, endocardial, and midmyocardial cells also referred to as midwall cells), sino-atrial node and Purkinje fibers by incorporating data of combinations of hERG and Nav1.5 channel subunit variants to cell models. The simulation used established electrophysiological models for atrial, ventricular cardiomyocytes, and Purkinje cells (Tomek et al., 2019; Inada et al., 2009; Trovato et al., 2020), respectively. We modified these established models to incorporate the concentration-dependent effects and variant-specific IC_50_ values of amiodarone. We selected Trovato’s Purkinje cell model because prior studies iteratively calibrated it against experimental data (Wu et al., 2008). Tomek’s model of the ventricular cardiomyocytes was used because it has updated chloride channel dynamics relative to the original O’Hara-Rudy (ORd) model (Tomek et al., 2020), and Inada’s model was the only model available for sino-atrial node (Amuzescu et al., 2021). Results from the simulation (ionic currents and membrane potential at given time points) were used to compute the drug cardiotoxicity metrics: APD90 and qNet (**Figure 2**).

### 2.1 Drug selection and concentration

Amiodarone, a widely used class III antiarrhythmic drug, is indicated for the treatment of recurrent ventricular fibrillation and recurrent hemodynamically unstable ventricular tachycardia (Kodama et al., 1997; Florek et al., 2024). It is used in this study because of its effect on both hERG and Nav1.5 channel subunits, its association with drug-induced TdP, and the availability of *in vitro* data on the drug’s IC_50_ values for various wild type (WT) and mutant alleles of hERG and Nav1.5 subunits. Different concentrations (C) of amiodarone were used to compute the cardiotoxicity metrics: [0, 1 x C_max_, 10 x C_max_, 20 x C_max_, 50 x C_max_, and 100 x C_max_] where C_max_ is the peak plasma concentration of the unbound drug at the recommended therapeutic dose, representing the fraction of the drug available to bind to ion channels. The C_max_ of amiodarone used in this study is 0.8 nM (Kramer et al., 2013).

### 2.2 Selection of ion channel subunit mutations and drug impact

The IC_50_ values of amiodarone on variants of hERG and Nav1.5 subunits were obtained from *in vitro* studies conducted by Zhang et al. (2016) and Jovianos-Santos et al (Joviano-Santos et al., 2021), respectively, and are shown in **Table 1**. The IC_50_ value of amiodarone for each subunit variant of hERG and Nav1.5 was used to compute the ionic current block for all combinations of wild-type and mutant channels. These specific variants were selected because IC_50_ data for amiodarone was available from published *in vitro* studies for both hERG and Nav1.5 variants, thereby incorporating experimentally derived values into a computational model. The selected Nav1.5 polymorphisms also reflect clinically meaningful population diversity. S524Y and S1103Y are present in 6% and 13% of African-descendent population respectively. R1193Q is present in 0.3% of Hispanics and V1951L is prevalent in 7% and 16% in White and Asian populations respectively (Joviano-Santos et al., 2021). In this work, genotype effects were parametrized by using variant-specific IC_50_ values only. Mutation-dependent changes in channel conductance, voltage-dependent gating, recovery kinetic, and trafficking were not explicitly modeled and therefore remain outside the scope of the current framework.

**Table 1:** IC_50_ values for Amiodarone on potassium and sodium channel subunits.

Alleles of sodium and potassium channels	IC_50_ (nM)			
hERG subunits				
WT	45
G648A	257
T623A	292.5
V659A	445.5
F656A	774
Y652A	913.5
S624A	981
Nav1.5 subunits				
WT	31700
R1193Q	77600
V1951L	93100
S1103Y	98600
S524Y	109800

### 2.3 Modeling amiodarone-induced current block

We modeled amiodarone’s impact on the single-cell action potential by selectively blocking specific sodium and potassium ionic currents. We calculated the fractional current block [1-β] for each amiodarone concentration using a Hill model, subsequently multiplying this fraction by the ionic current (I) to determine the total current blockade. It was then multiplied by the ionic current “I” to calculate the current blockade in the presence of amiodarone using Equation 1. The hill model has two key parameters: IC_50_ and the hill’s coefficient “h”. *In vitro* IC_50_ data for amiodarone on wild-type and mutated hERG and Nav1.5 channel subunits were obtained from prior studies by Zhang et al. (2016)and Joviano-Santos et al (Amuzescu et al., 2021). respectively, and hill’s coefficient, h, was fixed at 1 as in other studies (Mirams et al., 2011; Kramer et al., 2013; Mirams et al., 2014):

(1) $$\{\begin{array}{l} \beta =\frac{C^{h}}{IC50^{h}+C^{h}}\\ I_{drug}=\left(1-\beta \right)I \end{array}$$

### 2.4 Ionic currents

Ionic currents play a key role in regulating the action potential, each contributing to distinct phases of the process. For instance, fast sodium current (*I_Na_*), carried by Nav1.5 sodium channels, is crucial for the rapid depolarization phase, initiating the action potential. The rapid delayed rectifier potassium current *I_Kr_*, mediated by hERG channels, contributes to the repolarization phase, helping to terminate the action potential and reset the membrane potential. The simulation process consisted of computing the different ionic currents, using each electrophysiological model for cardiomyocytes.

We executed all simulations in MATLAB 2023b, utilizing a variable-order implicit method based on numerical differentiation formulas (NDF) suitable for efficiently solving stiff differential equations. The different electrophysiological models used for the project are available on cellml.org. Simulations were equilibrated by running 1000 beats at 1 Hz with the drug-induced channel block applied from the first beat, ensuring full stabilization of intracellular ion concentrations and gating variables under drug-equilibrated conditions. Prior to drug introduction, simulations were equilibrated by running 1000 beats at 1 Hz for each model to ensure full stabilization. Since the study focuses on the Nav1.5 and hERG channel subunits, the equations involving the *I_Na_* and *I_Kr_* were multiplied by their respective fractional current block to reflect the impact of amiodarone on those channels:

(2) $$I_{Na_{blocked}}=\left(1-\beta_{Na}\right)I_{Na}$$

(3) $$I_{Kr_{blocked}}=\left(1-\beta_{Kr}\right)I_{Kr}\;$$

#### 2.4.1 Ionic currents description for ventricular cardiomyocytes: endocardial, midmyocardial, and epicardial cells

This ionic current model is based on the work from Tomek et al (Tomek et al., 2019). The total ionic current for endocardial, midmyocardial and epicardial cells is the sum of the fast sodium current (*I_Na_*), the late sodium current (*I_NaL_*), the transient outward potassium current (*I_to_*), the L-type calcium current (*I_CaL_*), the sodium-calcium exchanger current (*I_CaNa_*), the calcium potassium current (*I_CaK_*), the rapid delayed rectifier potassium current (*I_Kr_*), the slow delayed rectifier potassium current (*I_Ks_*), the inward rectifier potassium current (*I_K_*_1_), the sodium-calcium exchanger current (*I_NaCa_*), the sodium-potassium pump current (*I_NaK_*), the background sodium current (*I_Nab_*), the background potassium current (*I_Kb_*), the potassium pump current (*I_pCa_*), the background calcium current (*I_Cab_*), the calcium-sensitive chloride current (*I_ClCa_*), and the background chloride current (*I_Clbk_*) (Equation 4).

(4) $$I_{ion}=I_{Na}+I_{NaL}+I_{to}+I_{CaL}+I_{CaNa}+I_{CaK}+I_{Kr}+I_{Ks}+I_{K1}+I_{NaCa}+I_{NaK}+I_{Nab}+I_{Kb}+I_{pCa}+I_{Cab}+I_{ClCa}+I_{Clbk}$$

##### 2.4.1.1 Ionic currents description for Purkinje cells

The ionic current model used for Purkinje cells is based on prior studies (Stewart et al., 2009; Trovato et al., 2020). The Trovato model for Purkinje cells was modified from the original Stewart model (Stewart et al., 2009) to include two additional Purkinje-specific currents: the T-type calcium current (I_CaT_) and the funny current (I_f_). The model we used defines the ionic current as the sum of the fast sodium current (*I_Na_*), the late sodium current (*I_NaL_*), the transient outward potassium current (*I_to_*), the L-type calcium current (*I_CaL_*), the sodium-calcium exchanger current (*I_CaNa_*), the calcium potassium current (*I_CaK_*), the rapid delayed rectifier potassium current (*I_Kr_*), the slow delayed rectifier potassium current (*I_Ks_*), the inward rectifier potassium current (*I_K_*_1_), the sodium-calcium exchanger current (*I_NaCa_*), the sodium-potassium pump current (*I_NaK_*), the background sodium current (*I_Nab_*), the background potassium current (*I_Kb_*), the potassium pump current (*I_pCa_*), the background calcium current (*I_Cab_*), the sustained current (I_sus_), the T-type calcium current (I_CaT_) and the funny current (I_f_) (Equation 5).

(5) $$I_{ion}=I_{Na}+I_{NaL}+I_{to}+I_{CaL}+I_{CaNa}+I_{CaK}+I_{Kr}+I_{Ks}+I_{K1}+I_{NaCa}+I_{NaK}+I_{Nab}+I_{Kb}+I_{pCa}+I_{Cab}+I_{sus}+I_{CaT}+I_{f}$$

##### 2.4.1.2 Ionic currents description for atrial cells

The ionic current model used for atrial cells is based on prior studies (Inada et al., 2009). This model considers atrial cells-specific additional currents, the background currents (I_bNa_ and I_bCa_), the sarcolemmal calcium pump current (I_CaP_), and the ultrarapid delayed rectifier potassium current (I_Kur_). This model defines the ionic current as the sum of the fast sodium current (*I_Na_*), the transient outward potassium current (*I_to_*), the L-type calcium current (*I_CaL_*), the sarcolemmal calcium pump current (I_CaP_), the rapid delayed rectifier potassium current (*I_Kr_*), the slow delayed rectifier potassium current (*I_Ks_*), the inward rectifier potassium current (*I_K_*_1_), the sodium-calcium exchanger current (*I_NaCa_*), the background currents in sodium and calcium respectively (I_bNa_ and I_bCa_) the sodium-potassium pump current (I_NaK_), and the ultrarapid delayed rectifier potassium current (I_Kur_). (Equation 6).

(6) $$I_{ion}=I_{Na}+I_{to}+I_{CaL}+I_{CaP}+I_{Kr}+I_{Ks}+I_{K1}+I_{NaCa}+I_{NaK}+I_{bNa}+I_{bCa}+I_{Kur}$$

### 2.5 Drug toxicity evaluation

The Action Potential Duration at 90% repolarization (APD90) and the total amount of ionic charge that flows through ion channels from the beginning to the end of the action potential (qNet) are used to evaluate drug toxicity. APD90 is extracted from the value of the action potential models. Those electrophysiological models for ventricular cardiomyocytes, Purkinje fibers, and atrial cells were calibrated by Tomek et al. (2019), Trovato et al. (2020), and Inada et al. (2009), respectively. In this work, each model was used in its published, experimentally validated formulation as the baseline. Pharmacological perturbations were introduced through IC50/Hill-type channel block, consistent with the approach already adopted in the original publications.

After equilibrating the models by running 1000 beats at 1 Hz prior to drug introduction, we ran pre-pacing simulations of 1000 beats at 1 Hz with the drug-induced channel block applied from the first beat to each model, ensuring full stabilization of intracellular ion concentrations and gating variables under drug-equilibrated conditions. This is in line with the calibration protocols described in the respective original studies. APD90 and qNet were extracted from the final beat of this pre-pacing train, which represents the drug-equilibrated steady-state response. qNet calculations were performed only for ventricular cardiomyocytes, as validated qNet thresholds for proarrhythmic risk are available for this cell type within the CiPA framework. These models were modified for this study as described in Equations 2, 3 to incorporate the IC_50_ values and the drug concentrations as inputs. At any given drug concentration, two IC_50_ values for amiodarone that correspond to alleles of the hERG and the Nav1.5 channels were used to compute ionic currents blockade from Equation 1. The validity of these modifications was assessed by comparison between implemented input (IC50) and expected outputs (APD 90). As an example, the hERG wild-type allele exhibits the lowest IC_50_ value among the tested variants, it should therefore induce the greatest fractional block at any given amiodarone concentration. This expected trend was confirmed as the hERG wild-type allele yields the longest APD90 values in all simulations, consistent with the well-established role of I_Kr_ reduction in action potential prolongation (Saxena et al., 2017; Burashnikov et al., 2021). Similar comparison was performed with High IC50 and Low IC50 values for both hERG and Nav 1.5 alleles results. These models were modified for this study as described in Equations 2, 3 to incorporate the IC_50_ values and the drug concentrations as inputs. At any given drug concentration, two IC_50_ values for amiodarone that correspond to alleles of the hERG and the Nav1.5 channels were used to compute ionic currents blockade from Equation 1. We computed APD90 using Equation 7 as follows:

(7) $$qNet=\int_{0}^{APD}I_{Net}\left(t\right)dt$$

Where V_90_ is the membrane potential at 90% repolarization, V_peak_ is the maximum membrane potential, and V_rest_ the potential at resting state. APD90 is the time when V_90_ was reached. Normal ranges for APD90 for ventricular cardiomyocytes are between 250–450 ms, for atrial cells between 200–400 ms, and for Purkinje fibers ~ 300 ms (Kane and Terracciano, 2017).

qNet is defined as the total amount of ionic charge that flows through ion channels from the beginning to the end of the AP. Because qNet is primarily established in ventricular cardiomyocytes electrophysiology models, qNet analyses in this study were restricted to ventricular cell types. qNet is a digital biomarker for evaluating the proarrhythmic risk of drugs that measures the net charge across the cardiac cell over the period of the action potential, considering both depolarizing and repolarizing currents (Dutta et al., 2017). qNet has been reported to be more accurate in predicting TdP risk compared to APD (Dutta et al., 2017). Higher qNet values generally indicate lower proarrhythmic risk whereas lower qNet values indicate higher proarrhythmic risk (Dutta et al., 2017). Clinically, the importance of qNet lies in its ability to identify TdP risk across a variety of cellular and genetic settings. qNet is calculated using Equation 8 as follows:

(8) $$V_{90}\;=\;V_{peak}\;-\;0.9\;\left(V_{peak}-V_{rest}\right)$$

Where APD is the action potential duration and I_Net_ the sum of six major currents: the late sodium current *I_NaL_*, the transient outward potassium current *I_to_*, the L-type calcium current *I_CaL_*, the rapid delayed rectifier potassium current *I_Kr_*, the slow delayed rectifier potassium current *I_Ks_*, and the inward rectifier potassium current *I_K_*_1_. qNet values are used to distinguish between high, intermediate, and low-risk drugs. We used the threshold values from a prior study (Li et al., 2019) as follows: threshold 1(value = 0.0689 μC/μF) where values above this threshold are considered low risk and threshold 2 (value = 0.0579 μC/μF) where values below this threshold are considered high risk. Values that fall between threshold 1–2 are considered intermediate risk.

### 2.6 Statistical analysis

Two statistical tests were used to analyze the results and identify significant differences. First, we used a categorical regression analysis to identify significant effects of the mutation and concentration on both APD90 and qNet results. The regression analysis was performed using the statsmodel Python library linear model regression. Furthermore, two additional regression steps were implemented to refine the results and identify the most significant parameters. Effects were considered significant when *p< 0.05.

Second, a paired t-test was utilized to identify significant differences between two specific groups. Groups were defined as the results obtained for specific hERG & Nav1.5 allele combinations at all amiodarone concentrations. For example, the test was used to compare the APD90 values of groups of hERG-WT & Nav1.5-WT alleles with the APD90 values of groups of hERG-V659A & Nav1.5-R1193Q mutations at all amiodarone concentrations. This test was applied on the results obtained for APD90 and qNet toxicity biomarkers. Differences were considered significant when *p< 0.05.

## 3 Results

The effect of different concentrations of amiodarone on the cardiac electrophysiology of wild-type and variants of both hERG and Nav1.5 channel subunits were simulated and assessed. Changes in action potential at 90% polarization (APD90) and net charge (qNet) were recorded on each of the five types of cardiac cells using six concentrations of amiodarone. The results are summarized in **Supplementary Tables 4**-**11**. For each cell type and amiodarone concentration profile, the APD90 and qNet values were calculated for each hERG and Nav1.5 allele combinations. Both were affected by the different combinations of alleles of hERG and Nav1.5, although the impact varied by cell type.

### 3.1 APD90 results for all cell types

**Figure 3:**
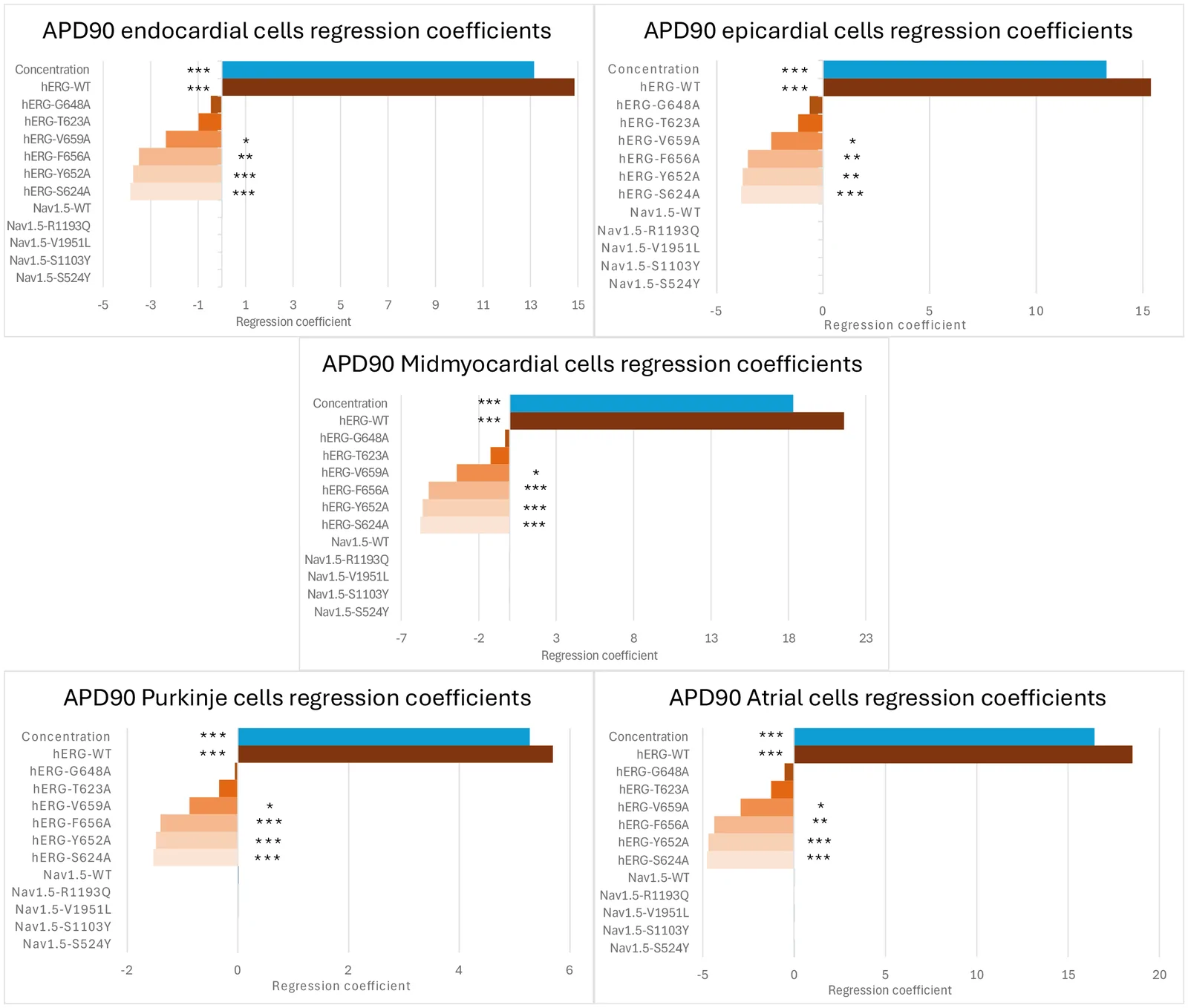
Result of the regression analysis for APD90 values obtained for each cell type. *: p-value <0.05; **: p-value <0.01; ***: p-value < 0.001.

Our simulations demonstrate that amiodarone consistently prolongs APD90 across all cell types compared to drug-free baselines and increases with rising drug concentrations across all allele combinations of hERG/Nav1.5 (**Supplementary Tables 4**-**7**). The results show that combinations of hERG-WT with different alleles of Nav1.5 exhibit the highest APD90 values for all cell types (**Supplementary Tables 4**-**7**). Given that the IC50 of amiodarone is the lowest for hERG-WT as shown in **Table 1**, the results are predominantly influenced by the hERG channel rather than Nav1.5. This is confirmed by the regression analysis that identified significant impacts of the hERG-WT, hERG-V659A, hERG-F656A, hERG-Y652A and hERG-S624A variants (**Figure 3**). Refined step wise regression confirmed the statistical impact from hERG-WT, hERG-F656A, hERG-Y652A and hERG-S624A, with the most impactful allele being the hERG-WT. Moreover, lower IC50 values of amiodarone indicate higher sensitivity and increased pharmacological potency.

**Figure 4:**
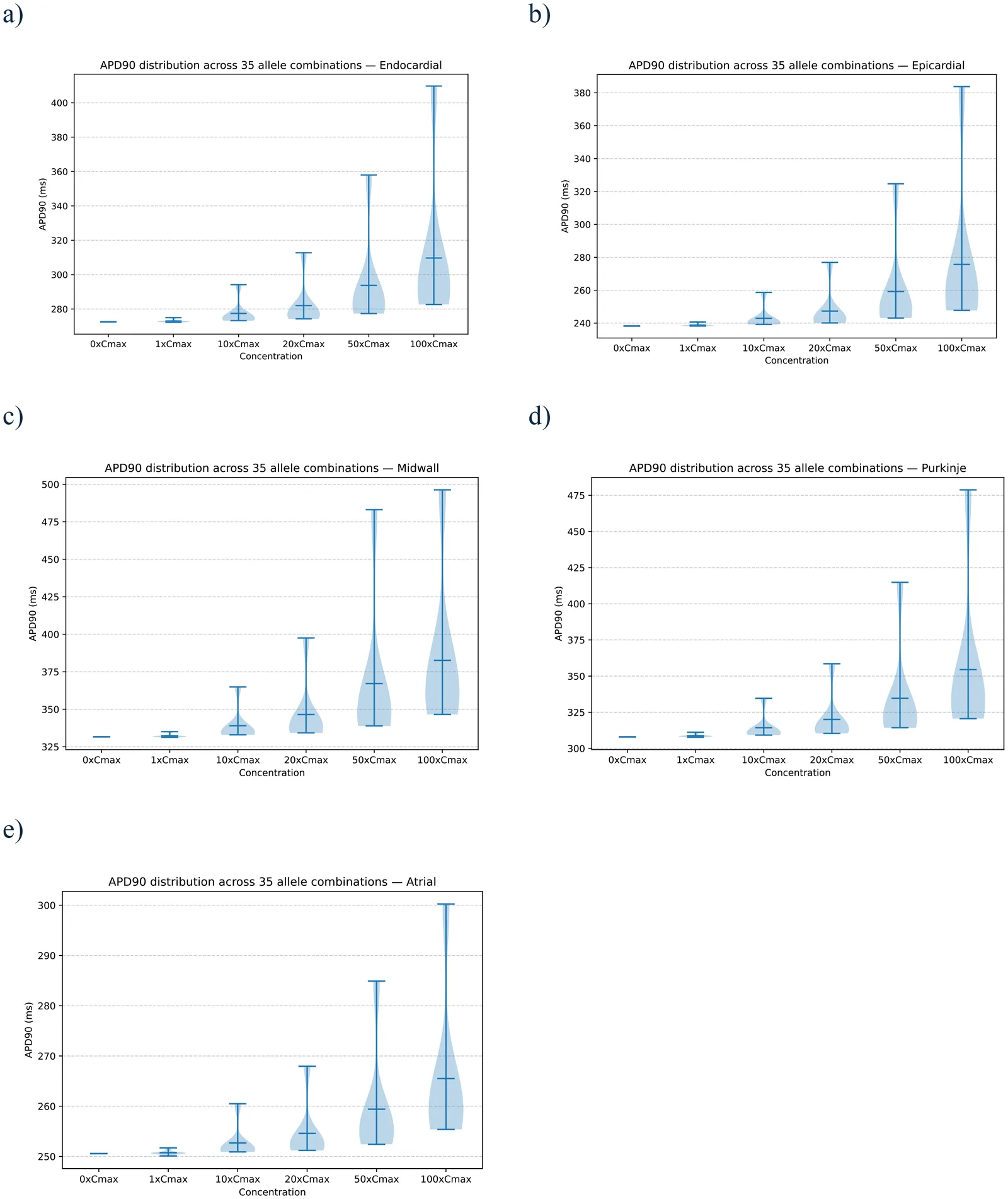
Violin plots for APD90 distribution across 35 allele combinations of hERG and Nav1.5 channels for: **(a)** Endocardial cells, **(b)** Epicardial cells, **(c)** Midmyocardial cells, **(d)** Purkinje cells, and **(e)** Atrial cells.

Furthermore, **Figure 4** provides a complementary representation of amiodarone’s concentration-dependent effects across all cell types. It illustrates the distribution and density of APD90 values across allele combinations at increasing amiodarone concentrations. The upper and lower bars of the plot indicate the highest and lowest APD90 values, the middle bar represents the mean value (**Supplementary Table 12**). The width of the plot reflects the density of observations whereby the broader regions reflect values shared by multiple allele combinations, while the narrower regions show less frequent variant-specific responses. A concentration-dependent increase in the mean values of APD90 is observed across all cell types, which indicates a progressive increase in repolarization duration with increasing amiodarone exposure. The mean APD90 values of all tested concentrations for ventricular cardiomyocytes and atrial cells remain within established physiological safety thresholds (approximately 250–450 ms and 200–400 ms, respectively). However, APD90 values for Purkinje rise above 300 ms, which is the safety threshold value, for the tested amiodarone concentrations (**Supplementary Table 12**). This indicates a prolonged repolarization phase and increased sensitivity to further drug-induced prolongation.

**Figure 5:**
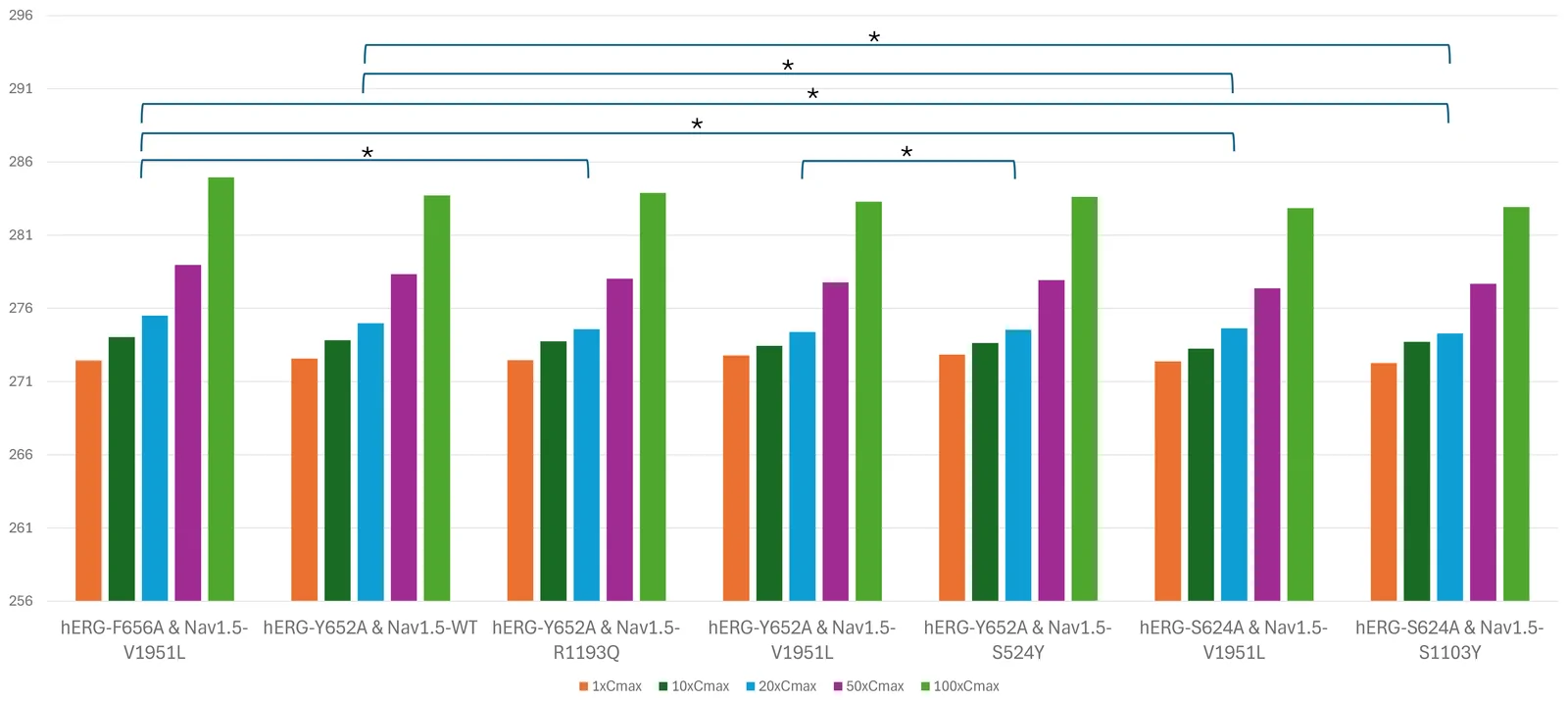
APD90 values obtained for endocardial cells at the tested concentration. *: p-value < 0.5.

**Figure 6:**
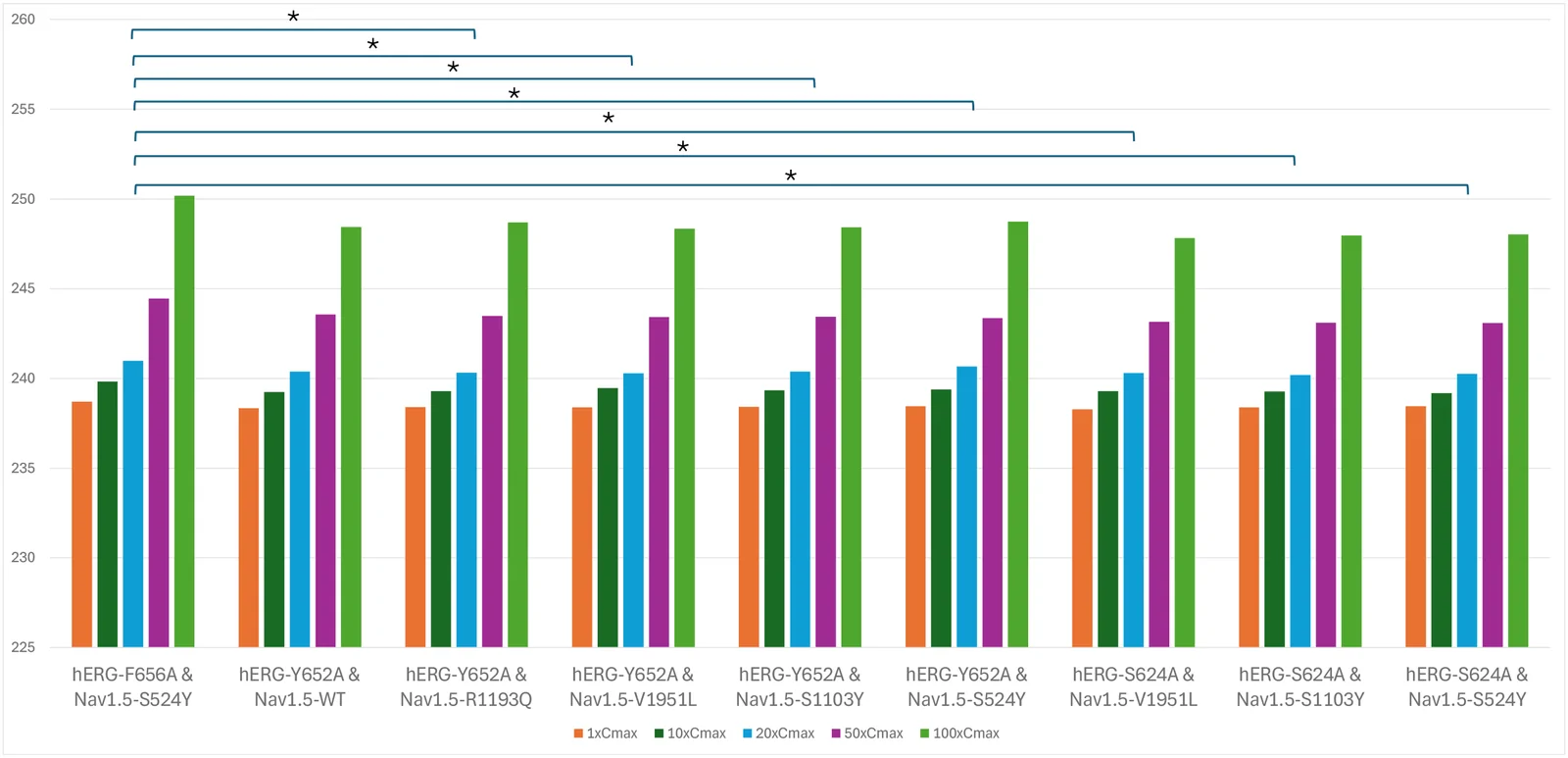
APD90 values obtained for epicardial cells at the tested concentration. *: p-value < 0.5.

**Figure 7:**
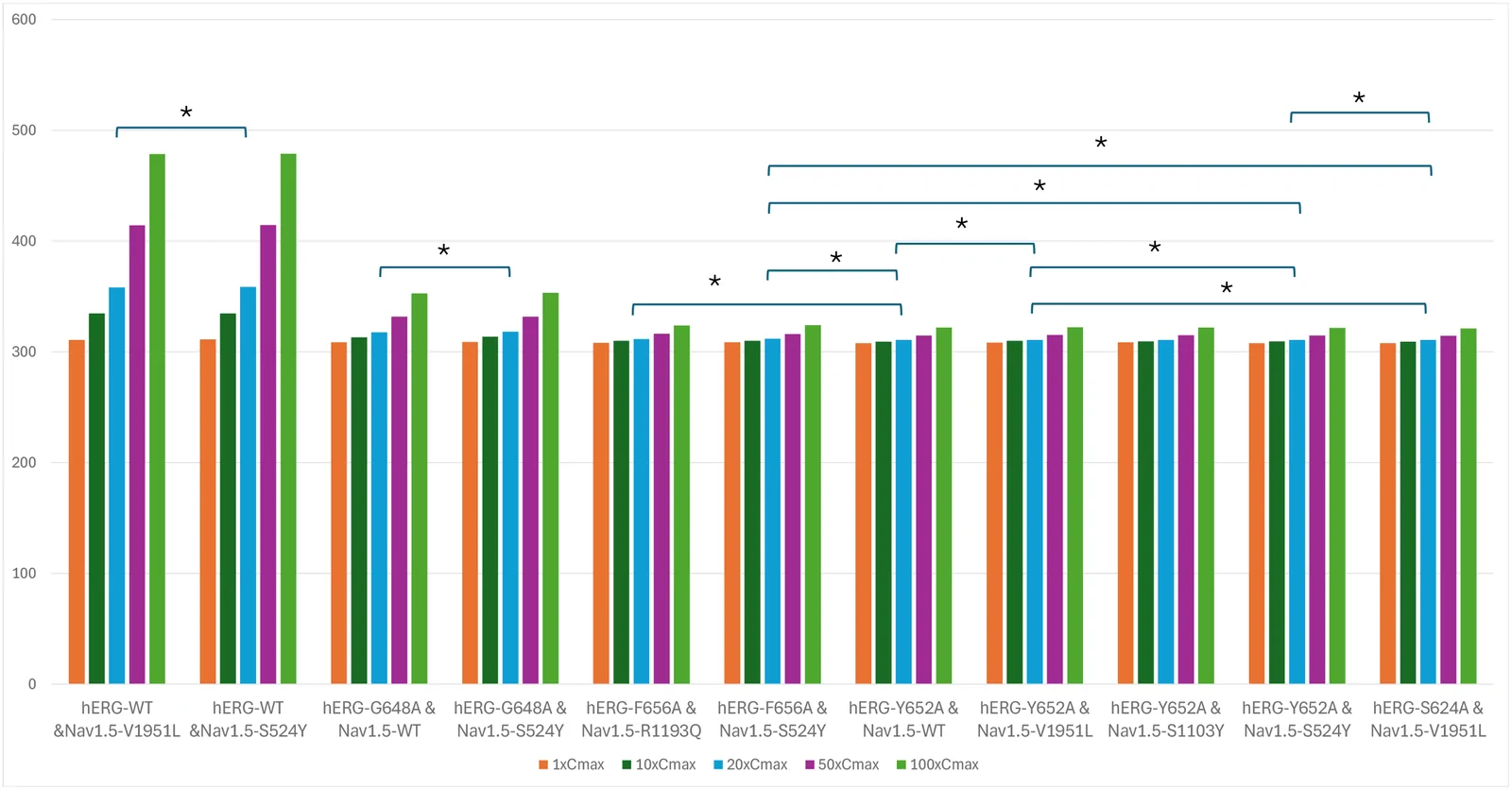
APD90 values obtained for Purkinje cells at the tested concentration. *: p-value < 0.5.

**Figure 8:**
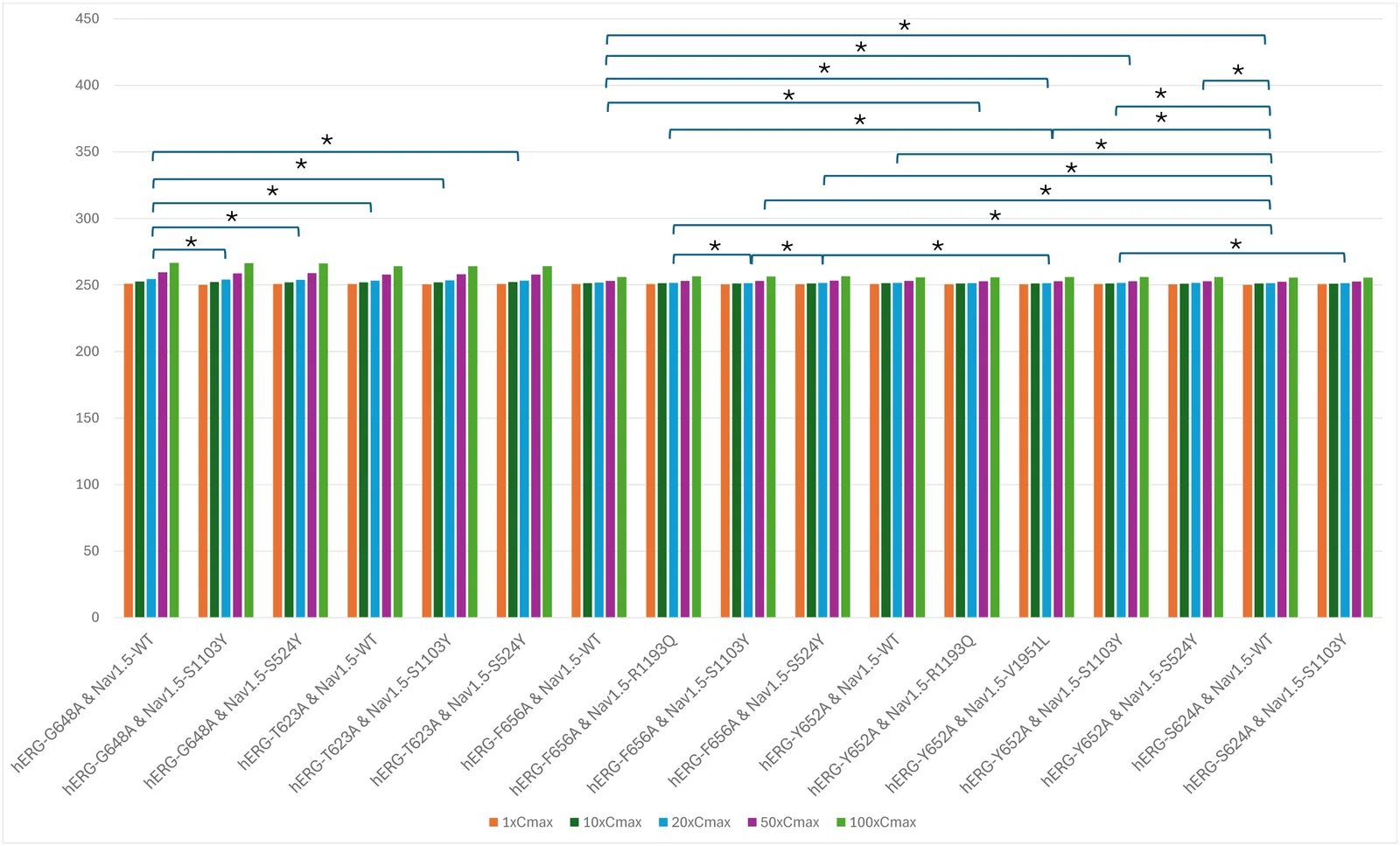
APD90 values obtained for atrial cells at the tested concentration. *: p-value < 0.5.

For all tested cell types, results showed significant statistical differences between hERG and Nav1.5 allele combinations across different cell types (except for midmyocardial cells which did not show statistical significance – results not shown) as outlined in **Figures 5**–**8** and **Table 2**. These differences suggest that cellular response to amiodarone is influenced by specific genetic variations in both hERG and Nav1.5 channels and that these effects are measurable across different channel combinations.

**Table 2:** Summary of APD90 results and statistically significant hERG&Nav1.5 alleles combinations across cardiac cell types.

Cell type	Statistically significant differences observed between combinations
Endocardial Cells	• hERG-F656A & Nav1.5-V1951L compared to hERG-Y652A & Nav1.5-R1193Q and hERG-S624A & Nav1.5-V1951L and hERG-S624A & Nav1.5-S1103Y. • hERG-Y652A & Nav1.5-WT compared to hERG-S624A & Nav1.5-V1951L and hERG-S624A & Nav1.5-S1103Y • hERG-Y652A & Nav1.5-V1951L and hERG-Y652A & Nav1.5-S524Y
Midmyocardial Cells	None
Epicardial Cells	• hERG-F656A & Nav1.5-S524Y compared to hERG-Y652A & Nav1.5-R1193Q and hERG-Y652A & Nav1.5-V1951L and hERG-Y652A & Nav1.5-S1103Y and hERG-Y652A & Nav1.5-S524Y and hERG-S624A & Nav1.5-V1951L and hERG-S624A & Nav1.5-S1103Y and hERG-S624A & Nav1.5-S524Y.
Purkinje Cells	• hERG-WT & Nav1.5-V1951L and hERG-WT & Nav1.5-S524Y • hERG-G648A & Nav1.5-WT and hERG-G648A & Nav1.5-S524Y • hERG-Y652A & Nav1.5-WT compared to hERG-F656A & Nav1.5-R1193Q and hERG-F656A & Nav1.5-S524Y and hERG-Y652A & Nav1.5-V1951L • hERG-F656A & Nav1.5-S524Y compared to hERG-Y652A & Nav1.5-S524Y and hERG-S624A & Nav1.5-V1951L • hERG-Y652A & Nav1.5-V1951L compared to hERG-Y652A & Nav1.5-S524Y and hERG-S624A & Nav1.5-V1951L • hERG-Y652A & Nav1.5-S524Y and hERG-S624A & Nav1.5-V1951L
Atrial Cells	• hERG-G648A & Nav1.5-WT compared to hERG-G648A & Nav1.5-S1103Y and hERG-G648A & Nav1.5-S524Y and hERG-T623A & Nav1.5-WT and hERG-T623A & Nav1.5-S1103Y and hERG-T623A & Nav1.5-S524Y • hERG-F656A & Nav1.5-WT compared to hERG-Y652A & Nav1.5-R1193Q and hERG-Y652A & Nav1.5-V1951L and hERG-Y652A & Nav1.5-S1103Y and hERG-S624A & Nav1.5-WT • hERG-F656A & Nav1.5-R1193Q compared to hERG-F656A & Nav1.5-S1103Y and hERG-Y652A & Nav1.5-V1951L and hERG-S624A & Nav1.5-WT • hERG-F656A & Nav1.5-S1103Y compared to hERG-F656A & Nav1.5-S524Y and hERG-S624A & Nav1.5-WT • hERG-F656A & Nav1.5-S524Y compared to hERG-Y652A & Nav1.5-V1951L and hERG-S624A & Nav1.5-WT • hERG-S624A & Nav1.5-WT compared to hERG-Y652A & Nav1.5-WT and hERG-Y652A & Nav1.5-V1951L • hERG-Y652A & Nav1.5-S1103Y compared to hERG-S624A & Nav1.5-WT and hERG-S624A & Nav1.5-S1103Y • hERG-Y652A & Nav1.5-S524Y compared to hERG-S624A & Nav1.5-WT

### 3.2 qNet results for endocardial, epicardial, and midmyocardial cells

**Figure 9:**
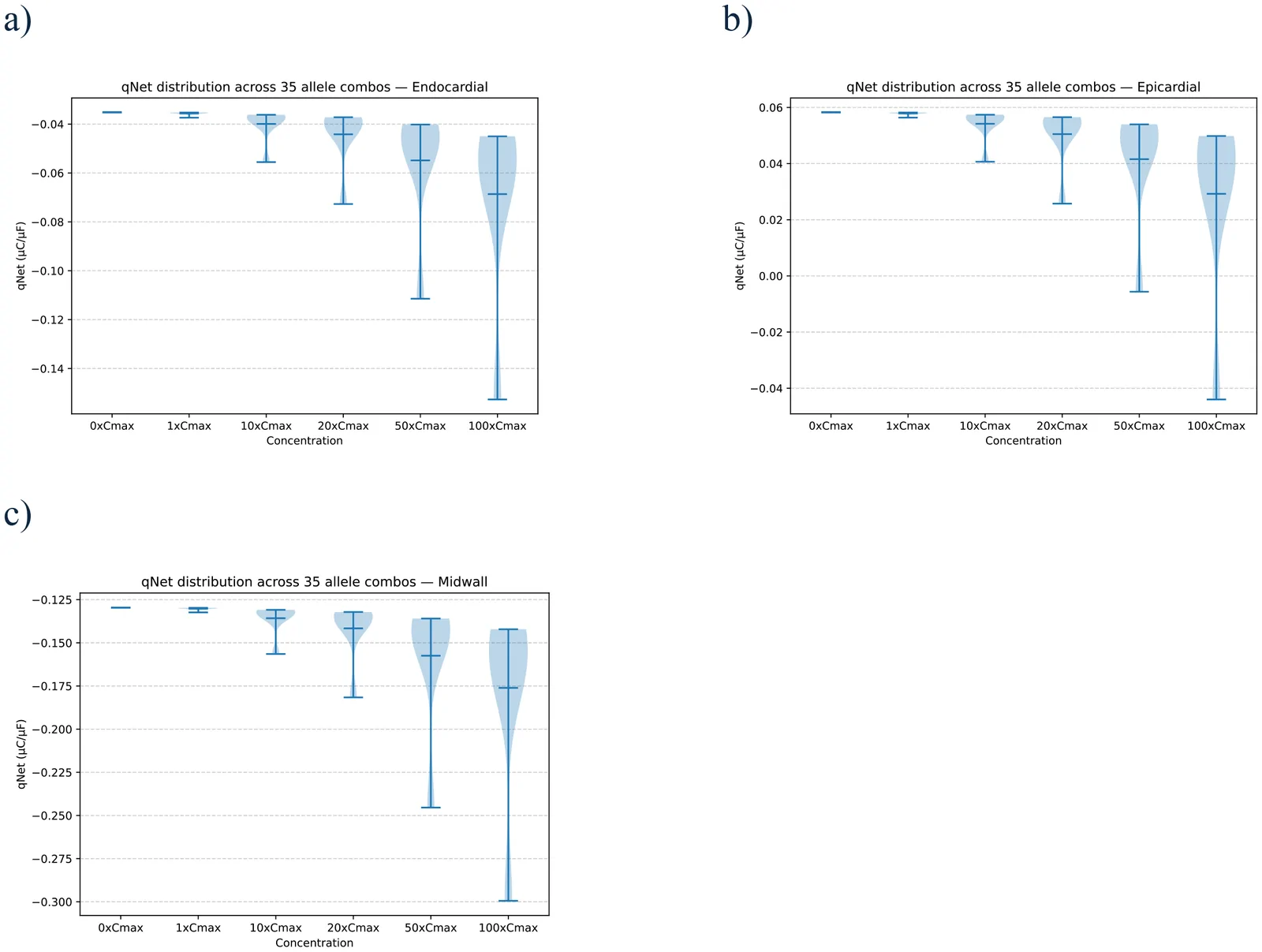
qNet distribution across 35 allele combinations of hERG and Nav1.5 channels for: **(a)** Endocardial cells, **(b)** Epicardial cells, and **(c)** Mid cells.

Similarly, we calculated the qNet values across endocardial, epicardial, and midmyocardial cells. Across all tested cell types, baseline qNet values peaked before drug exposure and exhibited a strict inverse relationship with amiodarone concentration. Combinations pairing the hERG-S624A mutation with Nav1.5 alleles yielded the highest qNet values across all amiodarone concentrations, indicating these specific genotypes confer the lowest arrhythmic risk. Conversely, the combination of hERG-WT with Nav1.5 alleles demonstrated the highest proarrhythmic potential, consistently producing the lowest qNet values at matched concentrations. Furthermore, violin plots illustrate how amiodarone alters qNet values across allele combinations in a dose-dependent manner as shown in **Figure 9**. The plots illustrate the ranges and mean values of qNet (**Supplementary Table 13**), while the plot width reflects the density of values thereby distinguishing between values shared by multiple combinations from less frequent values. Based on previously established qNet thresholds (0.0689 μC/μF for low risk and 0.0579 μC/μF for high risk), epicardial cells remain within the low to intermediate risk category at therapeutic concentration, while endocardial and particularly midmyocardial cells trend towards the high-risk category. Values that fall below the established safety threshold reflect reduced net repolarizing charge and diminished repolarization reserve, therefore increasing susceptibility to action potential prolongation and proarrhythmic events. Supratherapeutic amiodarone concentrations further decrease qNet values, particularly in midmyocardial cells, indicating concentration-dependent and cell-specific responses.

**Figure 10:**
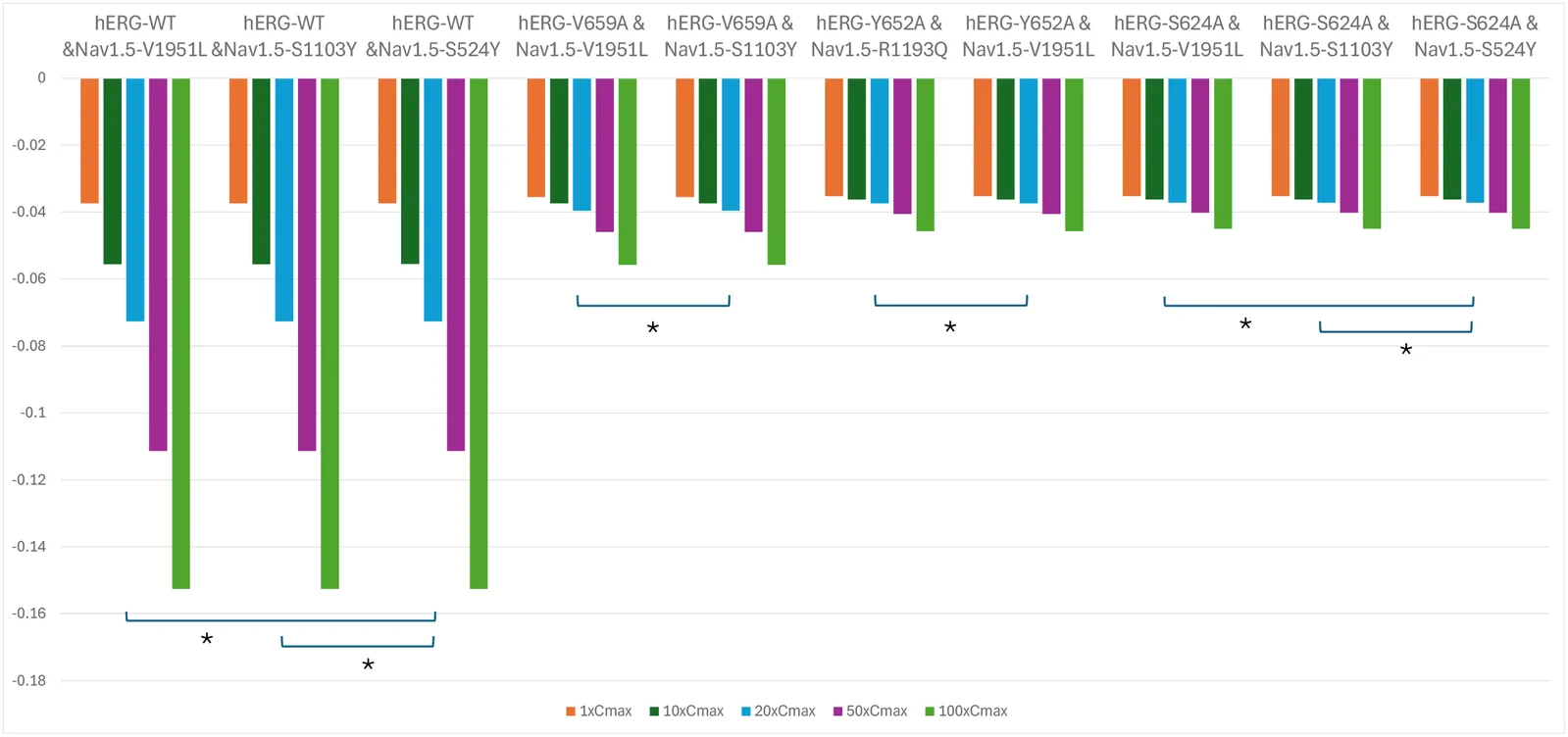
qNet values obtained for endocardial cells at the tested concentration. *: p-value < 0.5.

**Figure 11:**
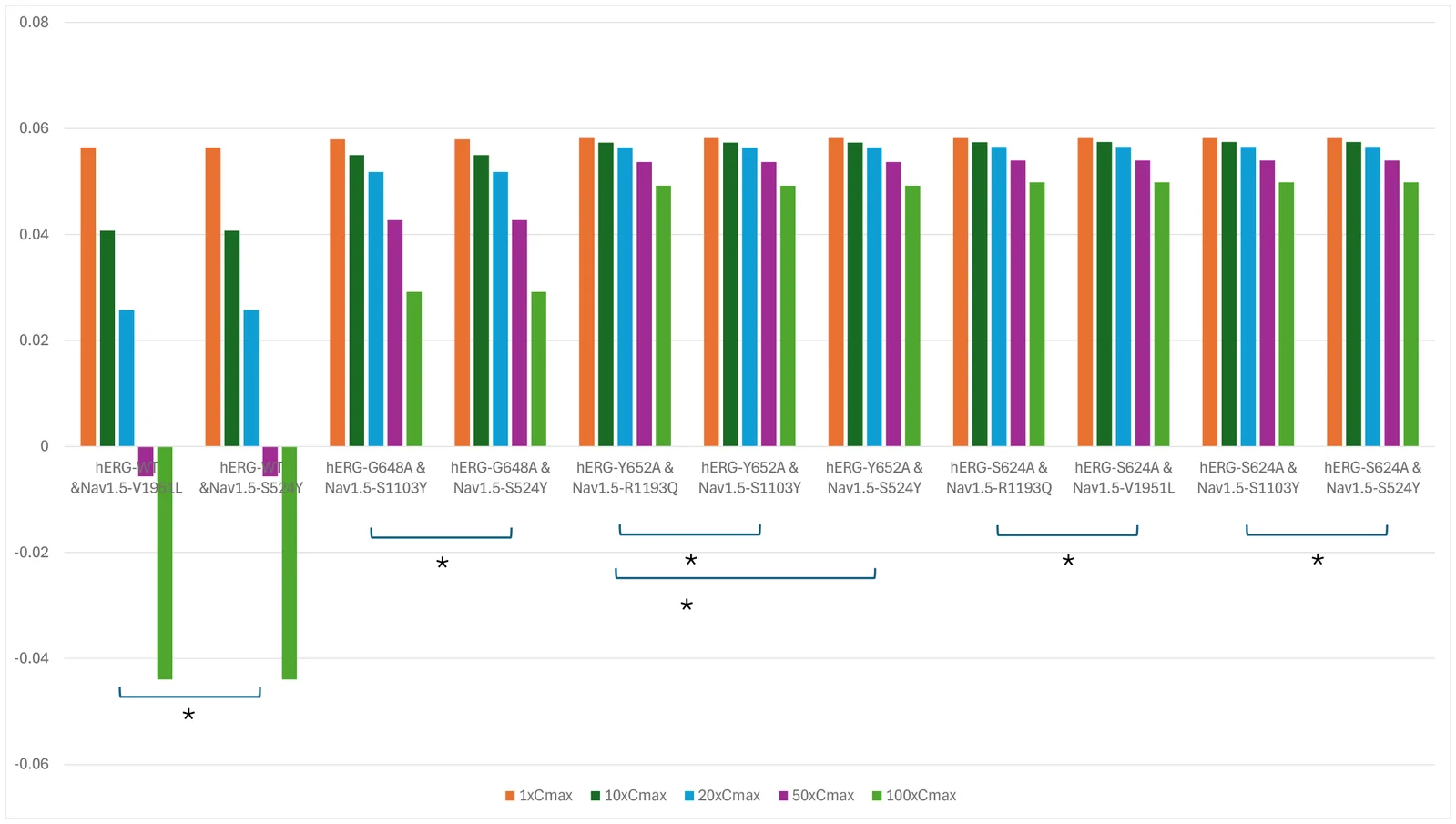
qNet values obtained for epicardial cells at the tested concentration. *: p-value < 0.5.

**Figure 12:**
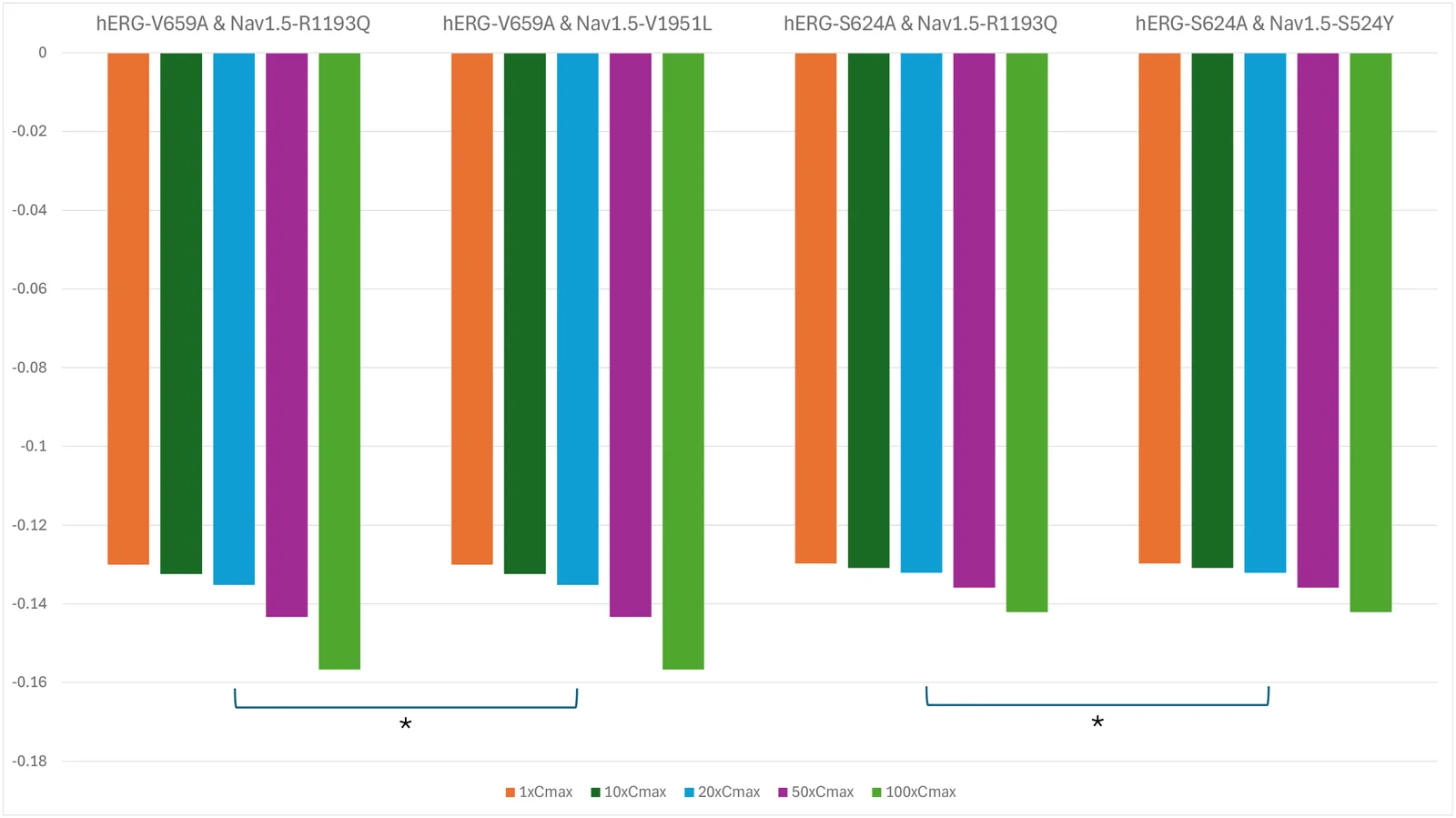
qNet values obtained for midmyocardial cells at the tested concentration. *: p-value < 0.5.

**Figure 13:**
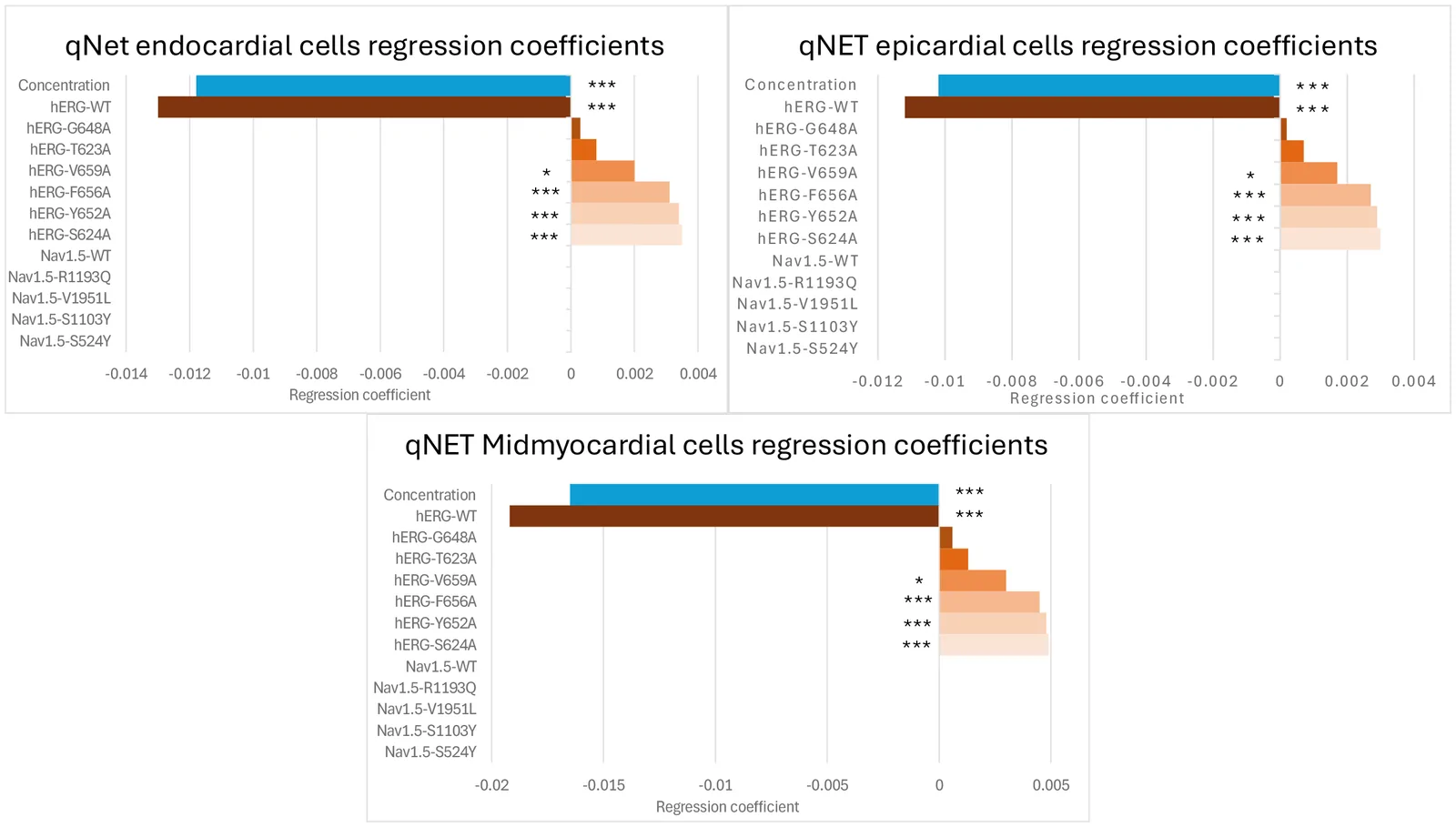
Result of the regression analysis for qNet values obtained for each cell type. *: p-value < 0.5, ***: p-value <0.001.

Amiodarone exposure exposed significant, variant-specific differences in qNet across all tested cell types (**Figures 10**–**12**; **Table 3**). This indicates that the presence of genetic mutations in subunits of cardiac ion channels among patients may alter their response to amiodarone thereby underscoring the need for personalized risk assessment. Similarly to the results from the APD90, the results are predominantly influenced by the hERG channel rather than Nav1.5. This is confirmed by the regression analysis that identified significant impact of hERG-WT, hERG-V659A, hERG-F656A, hERG-Y652A and hERG-S624A (**Figure 13**). Refined step wise regression showed a statistical impact from hERG-WT, hERG-F656A, hERG-Y652A and hERG-S624A, with the most impactful allele being the hERG-WT.

**Table 3:** Summary of qNet results and statistically significant hERG and Nav1.5 alleles combinations across cardiac cell types.

Cell type	Statistically significant differences observed between combinations
Endocardial Cells	• hERG-WT & Nav1.5-S524Y compared to hERG-WT & Nav1.5-V1951L and hERG-WT & Nav1.5-S1103Y • hERG-V659A & Nav1.5-V1951L compared to hERG-V659A & Nav1.5-S1103Y • hERG-Y652A & Nav1.5-R1193Q compared to hERG-Y652A & Nav1.5-V1951L • hERG-S624A & Nav1.5-S524 compared to hERG-S624A & Nav1.5-S1103Y and hERG-S624A & Nav1.5-V1951L
Midmyocardial Cells	• hERG-V659A & Nav1.5-R1193Q compared to hERG-V659A & Nav1.5-V1951L • hERG-S624A & Nav1.5-R1193Q compared to hERG-S624A & Nav1.5-S524Y
Epicardial Cells	• hERG-WT & Nav1.5-V1951L compared to hERG-WT & Nav1.5-S524Y • hERG-G648A & Nav1.5-S1103Y compared to hERG-G648A & Nav1.5-S524Y • hERG-Y652A & Nav1.5-R1193Q compared to hERG-Y652A & Nav1.5-S1103Y and hERG-Y652A & Nav1.5-S524Y • hERG-S624A & Nav1.5-R1193Q compared to hERG-S624A & Nav1.5-V1951L • hERG-S624A & Nav1.5-S1103Y compared to hERG-S624A & Nav1.5-S524Y

### 3.3 Integrated analysis of APD90 and qNet results across cardiac cell types

The endocardial cell results show a clear, allele-dependent modulation of APD90 and qNet, indicating that even minor shifts in ion channel drug sensitivity can produce measurable changes in repolarization. Given the endocardium’s role in initiating ventricular contraction, this tissue’s altered response under specific genetic variants could lead to subtle delays in conduction timing and contribute to arrhythmogenic risk. Importantly, these findings highlight how patient-specific alleles might amplify or mitigate drug effects, even in conventionally ‘average-risk’ tissues.

Epicardial cells showed moderate but distinct variation in response to drug exposure, especially across Nav1.5 variants. This layer, being the last to repolarize, contributes critically to transmural repolarization gradients. As such, slight prolongation in this region, if misaligned with changes in midmyocardial or endocardial layers, could exacerbate dispersion of repolarization, setting the stage for unidirectional block and re-entry circuits. These results support the use of cell-specific modeling approaches when evaluating proarrhythmic risk.

Midmyocardial cells results revealed the highest reduction in APD90 values across all combinations of hERG mutations and Nav1.5 alleles relative to the hERG-WT and Nav1.5 allele combinations among all cell types. Moreover, they demonstrated the greatest sensitivity to drug-induced APD90 prolongation. This aligns with their unique ionic current makeup, specifically low I_Ks_ and elevated I_NaL_, which predisposes them to prolonged repolarization and early afterdepolarizations under stress. Because midmyocardial depolarizations and repolarizations are often ‘averaged out’ in ECG readings, these risks remain hidden in clinical settings but are revealed in cell-specific models. This underscores the value of computational approaches for identifying latent arrhythmic substrates.

In Purkinje cells, values of APD90 at 1 x C_max_ of amiodarone for hERG- F656A, Y652A, and S624A are slightly lower than those recorded in the absence of the drug. The results also indicated a minimal effect of mutation combinations on amiodarone’s effects. However, despite only modest changes in APD90, Purkinje cells pose outsized arrhythmic risk due to their intrinsic automaticity and their role in conduction initiation. Certain allele combinations induced non-linear qNet changes, pointing to altered ionic balance in these highly excitable cells. These subtle changes, often invisible to QT-based assessments, may lower the threshold for triggered activity.

Among the cells analyzed, atrial cells demonstrated the smallest changes in APD90 and qNet in response to amiodarone, consistent with their reduced reliance on I_Kr_ and greater contribution from atrial-specific currents such as I_Kur_. Nonetheless, even these modest effects influence atrial fibrillation susceptibility in genetically predisposed individuals. Importantly, these findings underscore how the same ion channel mutation may function silently in one tissue type, such as the atrium, while inducing proarrhythmic risk in another, such as ventricular tissue. This further reinforces the importance of tissue-specific modeling for personalized safety assessments.

## 4 Discussion

In this study, we used a personalized in silico modeling framework to examine how amiodarone influences cardiac repolarization across five major cell types, ventricular endocardium, epicardium and midmyocardial, plus Purkinje and atrial cells, under different genetic backgrounds. We focused on 35 combinations of hERG and Nav1.5 alleles, evaluating APD90 across all cell types and qNet in ventricular cells under clinically relevant and supratherapeutic concentrations. To the best of our knowledge, this study presents the first systematic *in silico* simulation that considers various ion channel variants using a multichannel drug block context. Prior models mainly rely on IC_50_ averages of the drug in wild-type channels; however, our model integrates allele-specific variability in the channels across multiple cardiac cell types. While not exhaustive, this framework was designed to explore the extent to which genetic variations could affect drug sensitivity and can synergize with amiodarone block to influence repolarization dispersion and torsadogenic risk. This approach offers detailed insights that are otherwise difficult to uncover using population-averaged models alone.

Regression analysis showed that the drug concentration and hERG genotype were the main determinants of APD90 and qNet values (**Figures 3**, **13**). This suggests that within this present framework, amiodarone’s electrophysiological effects were mainly driven by hERG/I_Kr_ mediated differences in drug sensitivity, which is consistent with the established role of hERG inhibition in delaying repolarization and prolonging the duration of action potential. Additionally, prior work showed that amiodarone interacts with the hERG pore and that inhibition of repolarizing currents is a major determinant of APD-related proarrhythmic biomarkers (Kodama et al., 1997; Mirams et al., 2011; Zhang et al., 2016; Saxena et al., 2017; Burashnikov et al., 2021), which is consistent with the results of the regression analysis in this study. In parallel, results of the paired t-test analysis of specific hERG-Nav1.5 combinations showed significant differences across various hERG and Nav1.5 combinations, indicating that accounting for possible mutations in different channels is important for predicting the overall response of the drug.

This finding is expected given amiodarone’s multichannel blockade profile, and further reinforces the importance of the broader contribution of additional ion channels and currents in understanding the overall electrophysiological response to the drug (January and Riddle, 1989; Kodama et al., 1997; Wu et al., 2008).

The most consistent observation was the variability in response across the genetic background. For instance, combinations involving hERG-WT and certain Nav1.5 mutations consistently showed elevated APD90 (**Table 2**), particularly at higher drug doses which suggest a higher risk of proarrhythmia in individuals with these variants. In contrast, hERG-S624A combinations had shorter APD90 and higher qNet (**Table 3**), which aligns with prior work indicating this mutation weakens drug binding due to alterations in the selectivity filter (Zhang et al., 2016; Emigh Cortez et al., 2023). These results indicate that genetic context meaningfully alters how a patient might respond to drug-induced I_Kr_ block.

Additionally, we observed that tissue type further plays a role in modulating responses. Among the ventricular cells, midmyocardial cells were the most sensitive to APD90 prolongation (**Table 2**). This heightened vulnerability likely stems from their unique electrophysiological profile, which is characterized by reduced I_Ks_ and elevated late I_Na_ currents. This predisposes them to repolarization delay and early afterdepolarizations (EADs) (Liu and Antzelevitch, 1995; Zygmunt et al., 2001; Antzelevitch and Burashnikov, 2011). Although midmyocardial cells are known to be more susceptible to EADs when repolarization is delayed, EADs were not evaluated in the present simulations; therefore, the interpretation of risk in these cells is based on APD90 and qNet values rather than evidence of triggered activity. More broadly, our findings show that even small genotypic-specific variations in APD90 when combined with drug-induced blockade can intensify repolarization dispersion and heighten torsadogenic risk. In clinical settings, such subtle changes may not be apparent when measured by traditional surface ECGs until they become critical, but cell-specific in silico modeling allows us to capture their effect at the earliest onset.

Purkinje cells presented an interesting case. Although their APD90 shifts were modest, their baseline durations were long and their role in initiating impulses makes them a potentially overlooked contributor to arrhythmic risk. Interestingly, atrial cells were far less responsive to amiodarone, showing limited APD90 changes across genotypes and doses. This muted response is likely due to their reduced reliance on I_Kr_ and dominant I_Kur_-mediated repolarization (Courtemanche et al., 1999; Nattel, 2002; Wettwer et al., 2004; Burashnikov et al., 2008). Their shorter baseline APD90 may also constrain the extent of prolongation. Our collective findings suggest that a genotype-based approach to risk assessment should be adopted in situations involving multichannel block, especially for medications such as amiodarone, where the drug’s diverse target interactions may vary significantly among different genetic profiles. Therefore, integrating pharmacogenomic screening into preclinical or early clinical evaluations could enable the identification of genotypes that may be at risk before adverse reactions occur.

Our data strongly indicate that cellular context strictly dictates the manifestation of these mutation effects. The data in **Tables 2**, **3** suggest that some combinations had statistically significant effects on APD90 and qNet values in some cell types, but had little to no effects in others. This kind of context dependence suggests that genetic risk cannot be assessed in isolation, but rather it needs to be evaluated considering where and how that gene is expressed in the heart. Furthermore, including allele frequency data such as Nav1.5-S1103Y (13% in African-descendent populations), Nav1.5-S524Y (6% in African-descendent populations), Nav1.5-R1193Q (0.3% in Hispanics), and Nav1.5-V1951L (7% in Whites, 16% in Asians) makes these findings more clinically and demographically relevant. These population-specific polymorphisms suggest that individuals from specific ancestral backgrounds may be more susceptible to drug-induced QT prolongation or TdP. For instance, the increased prevalence of Nav1.5-S1103Y within individuals of African ancestry indicates that amiodarone-induced proarrhythmia might disproportionately impact this population. This demonstrates how genotype-informed simulations can identify high-risk individuals that might otherwise remain undetected by current safety screening protocols.

Perhaps most importantly, the simulations revealed substantial dispersion of repolarization across cell types under certain genotypes. Combinations involving hERG-WT with Nav1.5 mutations showed large differences in APD90 across tissues, a key finding that raises concerns about transmural dispersion in certain genotypes, which is a recognized foundation for TdP (Antzelevitch, 2001). Unlike average QT or APD metrics, this in silico modeling approach allows direct comparison of these differences across simulated cardiac cell types. This approach offers a clear benefit by modeling genotype-specific responses across distinct cardiac cell types, thereby advancing individualized cardiotoxicity prediction, in contrast to current CiPA models that rely on simple averages of IC_50_ values.

Mechanistically, many of these effects trace back to variation in drug-channel binding. We used IC_50_ values as a proxy for dissociation constant (K_d_) (DeMarco et al., 2019; Wang et al., 2022) and found that higher IC_50_ values, often seen in mutations such as hERG-S624A were correlated with reduced sensitivity to amiodarone’s blocking effects. These allele-specific differences were consistently reflected in both APD90 and qNet patterns across the ventricular tissues. Within this context, a key modeling simplification is that variant effects were represented through changes in IC_50_ values of amiodarone. This shows how experimentally observed differences in drug sensitivity across channel variants influence APD90 and qNet values. While mutations may contribute to other channel behaviors, such as channel conductance or gating kinetics, parametrizing drug effects through IC_50_ values remain a standard and widely used approach in *in silico* proarrhythmia modeling for simulation of biomarkers such as APD90 and qNet. Accordingly, these findings should be interpreted as showing how variant-specific differences in amiodarone sensitivity influence APD90 and qNet within this modeling framework. This means that the observed changes in APD90 and qNet should be interpreted specifically as the effect of variant-dependent amiodarone sensitivity, rather than as a complete representation of all mutation-dependent effects on channel function. The present framework is regarded as a pharmacology-driven model of variant-specific amiodarone response. Incorporating additional mutation-specific functional properties in future work could further strengthen the physiological relevance of this model. Taken together, our findings illustrate how combining genotype-level information with cell-specific modeling can uncover hidden risk profiles, some of which may not be evident in experimental models or ECG-based screening. This approach can support more informed safety assessments and help prioritize experimental validation of high-risk allele-drug interactions.

## 5 Conclusion, limitations, and future directions

This study introduces a multi-variant, cell-type-specific in silico modeling framework, used to evaluate the proarrhythmic potential of amiodarone across a range of hERG and Nav1.5 polymorphisms. While the results are illustrative rather than exhaustive, they highlight how genetic variation can substantially influence electrophysiological responses and by extension, clinical risk.

Our simulations revealed several important patterns. Some hERG and Nav1.5 combinations were associated with high APD90 and low qNet values (**Tables 2**, **3**), suggesting elevated proarrhythmic potential relative to other genetic combinations. Others, such as those involving hERG-S624A, exhibited reduced sensitivity to drug effects. Midmyocardial cells emerged as a particularly vulnerable tissue type, reinforcing the need for cell type specific evaluation in safety assessment. The data also highlighted how mutation effects vary across cardiac cell models, and that cell-type specific differences in APD90 and qNet may be a more informative marker of arrhythmogenicity beyond absolute prolongation alone.

This work builds on and extends the objectives of CiPA and FDA’s model-informed drug development (MIDD) framework by showing how in silico methods can be used to screen mutation–drug interactions in a patient-specific way. Despite the simplifications inherent to single-cell modeling, the consistency of APD90 and qNet trends supports the framework’s utility for hypothesis generation and early-stage safety evaluation.

Unlike current CiPA models that depend on population-averaged IC_50_ values from wild-type cardiac ion channels, our framework integrates genotype-specific variants. This integration allows for the simulation of inter-individual and population-specific responses, thereby addressing the limitations of CiPA’s current generalized approach. By linking pharmacogenomics to cardiac electrophysiology, this approach significantly advances CiPA’s predictive capacity from broad population-level assessment to genotype and patient specific risk evaluation. Furthermore, it establishes a clear pathway for incorporating pharmacogenomic data into future CiPA frameworks, aligning with the FDA’s emphasis on precision medicine.

There are several limitations worth noting. Our analysis was constrained by the availability of IC_50_ data, especially for Cav1.2 variants. The use of IC_50_ as a surrogate for the more fundamental property, Kd is an experimental simplification which makes it subject to measurement variability. In addition, the current framework represents genotype effects through static IC_50_ values and therefore focuses on altered drug sensitivity, without explicitly accounting for mutation-specific changes in channel conductance, voltage-dependent gating, or trafficking. Also, the omission of state- and time-dependent pharmacokinetics ignores the inherent dynamic nature of these processes that could lead to greater risk than would be predicted here. Additionally, simulating supratherapeutic concentrations up to 100× C_max_ may or may not reflect realistic localized clinical exposures, as single-cell models cannot capture variations due to conduction, mechanical effects, or arrhythmia initiation at the tissue level.

Furthermore, the current framework relies on static IC_50_ values and does not consider tissue- or organ-level validation; therefore, the spatial and mechanical complexities of cardiac electrophysiology are not portrayed. An important next step would be to evaluate whether the cell type specific heterogeneity observed in the present study translates into inter-tissue dispersion in tissue-level simulations. Future studies can resolve these limitations by integrating our framework with multi-scale digital twin architectures that include molecular dynamics, pharmacogenomics, and whole-heart simulations. Multi-scale models such as those from the Living Heart Project offer the possibility to scale this framework into a more physiologically detailed environment that capture local molecular and cellular behavior as well as simulating ECG, ejection fraction, and other clinically relevant readouts (Baillargeon et al., 2014; Sahli Costabal et al., 2018; Peirlinck et al., 2022; Aguado-Sierra et al., 2024).

Ultimately, we believe this type of modeling can support a new paradigm in drug safety: one that anticipates human variability and offers the potential to quantitate true population or individual risk. As regulatory agencies continue to support innovation in predictive modeling (Commissioner, 2018), and as genotyping becomes more routine, scalable in silico platforms such as this will become critical tools for more precise cardiotoxicity assessment, earlier risk detection, and better patient outcomes.

## Data Availability

The original contributions presented in the study are included in the article/**Supplementary Material**. Further inquiries can be directed to the corresponding authors.
