# Supplementary material for: Personalized in silico modeling of cardiac ion channel variants to predict drug-induced proarrhythmia risk
Source: Front Physiol. 2026 Aug 4;17:1828506. doi: 10.3389/fphys.2026.1828506 (PMC13495695; doi:10.3389/fphys.2026.1828506)
Supplement: Supplementary file 1 [file DataSheet1.pdf]

Personalized In Silico Modeling of Cardiac Ion Channel Variants to Predict Drug-Induced Proarrhythmia Risk

Alia Henedi♦1 & Jalal Cherkaoui♦2, Stelian Camara Dit Pinto[3], Steven M. Levine[4], Mohammed Cherkaoui3, Nicolas R. Gallo*[3] & Kenza E. Benzeroual*[1]

Supplementary material

The following tables (4-11) contain the data for hERG WT and variants arranged from the lowest to the highest IC_{50} value (from left to right, i.e.: hERG WT has the lowest IC_{50} value and hERG-S624A has the highest IC_{50} value). Similarly, for Nav1.5 WT and variants, the results in supplementary material tables (4-11) are arranged from the lowest IC_{50} value to the highest IC_{50} value (from top to bottom, i.e.: Nav1.5-WT has the lowest IC_{50} value and Nav1.5-S524Y has the highest IC_{50} value).

Table 4 - Heatmap-table displaying the APD90 (ms) values for endocardial cells at different amiodarone concentrations. Red color shows the highest APD90 values, whereas green color shows the lowest ones. Results in this table provide a comparison of all values relative to each other at one concentration.

APD90 values in ms for studied hERG and Nav1.5 alleles combinations							
0xCmax	hERG-WT	hERG-G648A	hERG-T623A	hERG-V659A	hERG-F656A	hERG-Y652A	hERG-S624A
Nav1.5-WT	272.5279651	272.5279651	272.5279651	272.5279651	272.5279651	272.5279651	272.5279651
Nav1.5-R1193Q	272.5279651	272.5279651	272.5279651	272.5279651	272.5279651	272.5279651	272.5279651
Nav1.5-V1951L	272.5279651	272.5279651	272.5279651	272.5279651	272.5279651	272.5279651	272.5279651
Nav1.5-S1103Y	272.5279651	272.5279651	272.5279651	272.5279651	272.5279651	272.5279651	272.5279651
Nav1.5-S524Y	272.5279651	272.5279651	272.5279651	272.5279651	272.5279651	272.5279651	272.5279651
1xCmax							
Nav1.5-WT	274.5494020	273.1141158	272.7516706	272.6388626	272.3853570	272.5635693	272.5397923
Nav1.5-R1193Q	274.9919623	272.7142869	273.1108117	272.7397139	272.8861141	272.4573691	272.8496140
Nav1.5-V1951L	274.5508047	272.9311015	272.8904750	272.8122193	272.4383404	272.7796492	272.3801941
Nav1.5-S1103Y	274.9070165	273.1423589	272.7520390	272.5210441	272.3546539	272.3793171	272.2686314
Nav1.5-S524Y	274.7715378	273.0870881	273.1479095	272.9175514	272.3527785	272.8309849	272.7650402
10xCmax							
Nav1.5-WT	293.6491015	276.4186314	276.1666929	274.4634621	273.8010929	273.8171820	273.5331154
Nav1.5-R1193Q	293.5929346	276.6694833	276.3055877	274.7662941	273.7176450	273.7421241	273.4257269
Nav1.5-V1951L	294.1477176	276.3163148	275.7723332	274.5647218	274.0292773	273.4456841	273.2404679
Nav1.5-S1103Y	293.5464874	276.3073338	276.3581738	274.7007126	273.9285745	273.7313965	273.7041234
Nav1.5-S524Y	294.1452739	276.2092131	276.1253447	274.5676862	273.8875840	273.6354181	273.2196110
20xCmax							
Nav1.5-WT	312.6087154	280.1295760	279.2449872	277.1370821	275.3592068	274.9710087	274.8602835
Nav1.5-R1193Q	312.7445391	280.6281982	279.5679768	277.0613264	274.9724608	274.5650743	274.3680056
Nav1.5-V1951L	312.5457120	280.5111583	279.3577632	277.3061435	275.5045271	274.3861142	274.6297035
Nav1.5-S1103Y	312.3394123	280.4195807	279.2927360	276.8932320	275.4003516	274.8829658	274.2796937
Nav1.5-S524Y	312.6985989	280.0098404	279.1091677	277.3227821	275.2435100	274.5350113	274.6013057
50xCmax							
Nav1.5-WT	357.4337105	291.3937972	289.4076762	283.9490214	279.2844240	278.3352052	277.7232751
Nav1.5-R1193Q	357.7022344	291.3866822	288.8889834	283.3847212	278.6869860	278.0223706	277.4378820
Nav1.5-V1951L	357.9897801	291.3048829	289.4714451	283.5374439	278.9658068	277.7672762	277.3594799
Nav1.5-S1103Y	357.5711726	291.2521002	288.8944013	283.5778599	279.1650333	278.1869070	277.6749696
Nav1.5-S524Y	357.4424277	291.6695761	288.8736452	283.6480502	278.7201491	277.9190704	277.5878564
100xCmax							
Nav1.5-WT	409.7272745	308.0603737	304.3086437	294.1826870	285.6547647	283.7000336	282.7001632
Nav1.5-R1193Q	409.1721807	307.9384085	304.5385212	294.1303570	285.4706074	283.8773188	282.6299842
Nav1.5-V1951L	409.4832051	308.6077688	304.3294833	294.0688612	284.9393611	283.2820822	282.8473537
Nav1.5-S1103Y	409.2469979	308.1781295	304.5534087	293.7711587	285.5418032	283.1093488	282.9109663
Nav1.5-S524Y	409.2639333	308.2474287	303.9297937	293.8470582	285.3716041	283.6120825	282.8849734

Table 5 - Heatmap-table displaying APD90 values (ms) for epicardial cells at various amiodarone concentrations. Red color red shows the highest APD90 values, whereas green shows the lowest ones. Results in this table provide a comparison of all values relative to each other at one concentration.

APD90 values in ms for studied hERG and Nav1.5 alleles combinations							
0xCmax	hERG-WT	hERG-G648A	hERG-T623A	hERG-V659A	hERG-F656A	hERG-Y652A	hERG-S624A
Nav1.5-WT	238.292620	238.292620	238.292620	238.292620	238.292620	238.292620	238.292620
Nav1.5-R1193Q	238.292620	238.292620	238.292620	238.292620	238.292620	238.292620	238.292620
Nav1.5-V1951L	238.292620	238.292620	238.292620	238.292620	238.292620	238.292620	238.292620
Nav1.5-S1103Y	238.292620	238.292620	238.292620	238.292620	238.292620	238.292620	238.292620
Nav1.5-S524Y	238.292620	238.292620	238.292620	238.292620	238.292620	238.292620	238.292620
1xCmax							
Nav1.5-WT	240.259206	238.623229	238.891590	238.442146	238.288546	238.342846	238.385277
Nav1.5-R1193Q	240.695858	238.615046	238.903997	238.521793	238.435218	238.412157	238.685993
Nav1.5-V1951L	240.327504	238.593725	238.520454	238.455279	238.237295	238.387298	238.278289
Nav1.5-S1103Y	240.674186	238.665112	238.495140	238.348293	238.404326	238.428337	238.394373
Nav1.5-S524Y	240.429030	238.538155	238.798068	238.534878	238.700864	238.450702	238.453048
10xCmax							
Nav1.5-WT	258.429599	241.993989	241.582891	240.264078	239.389731	239.242781	239.272753
Nav1.5-R1193Q	258.420979	242.165775	241.402746	240.614103	239.819948	239.284983	239.531286
Nav1.5-V1951L	258.686612	241.980691	241.415787	240.331685	239.569419	239.458462	239.293000
Nav1.5-S1103Y	258.321080	241.891936	241.411549	240.619681	239.519039	239.344010	239.273272
Nav1.5-S524Y	258.398027	242.295947	241.415481	240.326913	239.831430	239.385864	239.180165
20xCmax							
Nav1.5-WT	276.904884	245.881412	244.673085	242.489359	240.644245	240.385095	240.257625
Nav1.5-R1193Q	276.692134	245.482877	244.723857	242.671190	240.978748	240.320777	240.113175
Nav1.5-V1951L	276.866222	245.869674	244.844566	242.422420	240.674350	240.296070	240.307522
Nav1.5-S1103Y	276.684032	245.509797	244.672410	242.569733	240.855419	240.381791	240.200525
Nav1.5-S524Y	276.683693	245.575759	245.031712	242.815625	240.978874	240.669605	240.250322
50xCmax							
Nav1.5-WT	324.240404	255.951141	253.934329	248.739917	244.468783	243.569954	243.382920
Nav1.5-R1193Q	324.689680	256.009537	253.938634	248.639927	244.332660	243.479586	243.108706
Nav1.5-V1951L	324.627193	256.049118	254.044115	249.000861	244.467136	243.422467	243.161376
Nav1.5-S1103Y	324.249696	256.371539	253.903277	248.726472	244.263061	243.433171	243.101785
Nav1.5-S524Y	324.145208	256.009034	253.961441	248.894841	244.458302	243.353251	243.087660
100xCmax							
Nav1.5-WT	383.292611	272.494084	268.404585	258.537335	250.155302	248.436226	247.913586
Nav1.5-R1193Q	383.626891	272.456215	268.380275	258.674101	250.290630	248.697820	247.756007
Nav1.5-V1951L	383.371738	272.561396	268.379954	258.818000	250.154760	248.352891	247.818975
Nav1.5-S1103Y	383.578792	272.277920	268.380373	258.742569	250.242549	248.433114	247.963705
Nav1.5-S524Y	383.798833	272.351912	268.576868	258.748759	250.185634	248.741369	248.036119
