# Supplementary material for: Personalized in silico modeling of cardiac ion channel variants to predict drug-induced proarrhythmia risk
Source: Front Physiol. 2026 Aug 4;17:1828506. doi: 10.3389/fphys.2026.1828506 (PMC13495695; doi:10.3389/fphys.2026.1828506)
Supplement: Supplementary file 2 [file DataSheet2.pdf]

Table 8 - Heatmap-table displaying APD90 values (ms) for atrial cells at various amiodarone concentrations. Red color shows the highest APD90 values, whereas green shows the lowest ones. Results in this table provide a comparison of all values relative to each other at one concentration.

APD90 values in ms for studied hERG and Nav1.5 alleles combinations

0xCmax	hERG-WT	hERG-G648A	hERG-T623A	hERG-V659A	hERG-F656A	hERG-Y652A	hERG-S624A
Nav1.5-WT	250.5832338	250.5832338	250.5832338	250.5832338	250.5832338	250.5832338	250.5832338
Nav1.5-R1193Q	250.5832338	250.5832338	250.5832338	250.5832338	250.5832338	250.5832338	250.5832338
Nav1.5-V1951L	250.5832338	250.5832338	250.5832338	250.5832338	250.5832338	250.5832338	250.5832338
Nav1.5-S1103Y	250.5832338	250.5832338	250.5832338	250.5832338	250.5832338	250.5832338	250.5832338
Nav1.5-S524Y	250.5832338	250.5832338	250.5832338	250.5832338	250.5832338	250.5832338	250.5832338
1xCmax							
Nav1.5-WT	251.4255903	250.9111854	250.7357122	250.8462351	250.6564825	250.6414708	250.1009553
Nav1.5-R1193Q	251.7089629	250.3348819	250.7562680	250.6666553	250.6195544	250.5573896	250.7923400
Nav1.5-V1951L	251.3243966	250.9117549	250.8870563	250.6101157	250.6232720	250.4156145	250.6508135
Nav1.5-S1103Y	251.4637273	250.0966915	250.5760736	250.2805888	250.4820887	250.7597477	250.8072960
Nav1.5-S524Y	251.7123711	250.6668906	250.6955268	250.7163934	250.4820475	250.4829954	250.7370253
10xCmax							
Nav1.5-WT	260.4562884	252.5730809	251.8957372	251.4505334	251.2903431	251.2614666	251.0217099
Nav1.5-R1193Q	260.4936900	252.0797944	252.1053647	251.1314844	251.2720155	251.1995325	251.1277737
Nav1.5-V1951L	259.6428866	252.1654231	251.5592306	251.1309930	250.9505053	251.0449956	251.0041884
Nav1.5-S1103Y	259.5573166	252.1033924	251.9273224	251.5682564	251.1177504	251.1986347	250.9167025
Nav1.5-S524Y	260.3110205	251.9161212	252.1164670	251.6880502	251.1916604	251.0055524	250.9464036
20xCmax							
Nav1.5-WT	267.9519864	254.4239924	253.2115008	252.5081576	251.8375465	251.5565137	251.2316590
Nav1.5-R1193Q	267.8998028	254.3958224	253.2798404	252.4853192	251.4607195	251.2770916	251.2070116
Nav1.5-V1951L	267.9212636	254.1414104	254.0411103	252.2494707	252.0353272	251.3201636	251.3458613
Nav1.5-S1103Y	267.8479746	253.9985243	253.3333079	252.4196688	251.4042959	251.6139896	251.2475007
Nav1.5-S524Y	267.8048768	253.7512122	253.1684786	252.1553620	251.5793215	251.6114682	251.2042288
50xCmax							
Nav1.5-WT	284.2892073	259.4834733	257.8289278	256.0543183	253.0564901	252.9178515	252.4258765
Nav1.5-R1193Q	284.8003568	258.6102486	257.7744385	255.8315916	253.0482628	252.7887505	252.5210245
Nav1.5-V1951L	284.9015497	258.5908106	257.9914397	255.6646414	253.8991284	252.7158548	252.9278658
Nav1.5-S1103Y	284.8227006	258.6442429	257.9786040	255.9767658	253.0498676	252.7115226	252.5425621
Nav1.5-S524Y	284.2335419	258.7838520	257.8992249	255.7401897	253.1533096	252.7546475	253.1276000
100xCmax							
Nav1.5-WT	300.1910482	266.5351702	264.1779032	259.8487182	256.0007245	255.7643960	255.6163514
Nav1.5-R1193Q	300.0047782	266.6165164	264.0331552	260.6692525	256.4815904	255.7114832	255.6159818
Nav1.5-V1951L	300.2453056	266.3970931	264.3285186	259.8585115	256.3990309	255.8501802	255.4813544
Nav1.5-S1103Y	300.2167388	266.4215110	264.1176784	260.5328253	256.2732126	255.8543424	255.5042068
Nav1.5-S524Y	300.1764161	266.2722143	264.1095020	259.7311363	256.4708667	255.8920362	255.3863464

Table 9 - Heatmap-table displaying qNet values (μC/μF) for endocardial cells at various amiodarone concentrations. Red color shows the lowest qNet values, whereas green color shows the highest values. Results in this table provide a comparison of all values relative to each other at one concentration.

qNet values for studied hERG and Nav1.5 alleles combinations							
0xCmax	hERG-WT	hERG-G648A	hERG-T623A	hERG-V659A	hERG-F656A	hERG-Y652A	hERG-S624A
Nav1.5-WT	-0.035170068	-0.035170068	-0.035170068	-0.035170068	-0.035170068	-0.035170068	-0.035170068
Nav1.5-R1193Q	-0.035170068	-0.035170068	-0.035170068	-0.035170068	-0.035170068	-0.035170068	-0.035170068
Nav1.5-V1951L	-0.035170068	-0.035170068	-0.035170068	-0.035170068	-0.035170068	-0.035170068	-0.035170068
Nav1.5-S1103Y	-0.035170068	-0.035170068	-0.035170068	-0.035170068	-0.035170068	-0.035170068	-0.035170068
Nav1.5-S524Y	-0.035170068	-0.035170068	-0.035170068	-0.035170068	-0.035170068	-0.035170068	-0.035170068
1xCmax							
Nav1.5-WT	-0.03738202	-0.035561152	-0.035512446	-0.035395858	-0.035299528	-0.035280584	-0.03527284
Nav1.5-R1193Q	-0.037382556	-0.035560564	-0.03551466	-0.03539449	-0.035299295	-0.035280278	-0.035273501
Nav1.5-V1951L	-0.037383286	-0.035561272	-0.035512902	-0.035395818	-0.035298962	-0.035279277	-0.035273751
Nav1.5-S1103Y	-0.03738252	-0.035561763	-0.035512045	-0.035394765	-0.035299451	-0.035278394	-0.035273431
Nav1.5-S524Y	-0.037381959	-0.035561317	-0.03551384	-0.035395299	-0.035300148	-0.035278639	-0.035271466
10xCmax							
Nav1.5-WT	-0.055548478	-0.039028727	-0.038559236	-0.037410528	-0.036464992	-0.036269693	-0.036193898
Nav1.5-R1193Q	-0.055544816	-0.039025535	-0.038557967	-0.037405393	-0.036463355	-0.036268187	-0.036192088
Nav1.5-V1951L	-0.0555444	-0.039024821	-0.038557302	-0.037406919	-0.036464096	-0.0362662	-0.036192271
Nav1.5-S1103Y	-0.055546048	-0.039024135	-0.038557037	-0.037405832	-0.036462586	-0.036266073	-0.036192317
Nav1.5-S524Y	-0.055543451	-0.039024431	-0.038557648	-0.037406789	-0.03646186	-0.03626515	-0.036191335
20xCmax							
Nav1.5-WT	-0.07269785	-0.042760282	-0.041853844	-0.039607505	-0.037748187	-0.037360167	-0.037210987
Nav1.5-R1193Q	-0.072689256	-0.042753314	-0.041846916	-0.039599921	-0.037743404	-0.037353941	-0.037205807
Nav1.5-V1951L	-0.072690914	-0.042752585	-0.041847739	-0.039599899	-0.037741782	-0.037353288	-0.037204526
Nav1.5-S1103Y	-0.072690163	-0.04275266	-0.041847481	-0.0395993	-0.037740245	-0.037355061	-0.037204828
Nav1.5-S524Y	-0.07268916	-0.042755501	-0.0418467	-0.039600702	-0.037742143	-0.037354807	-0.037204355
50xCmax							
Nav1.5-WT	-0.111439754	-0.053260073	-0.051189104	-0.045952081	-0.041510175	-0.040569477	-0.040205618
Nav1.5-R1193Q	-0.11142315	-0.053246096	-0.051173813	-0.045936275	-0.04149601	-0.040555118	-0.040192492
Nav1.5-V1951L	-0.111419185	-0.053243684	-0.051171203	-0.045933972	-0.041493248	-0.04055252	-0.040191258
Nav1.5-S1103Y	-0.111418959	-0.0532438	-0.05117149	-0.045931621	-0.04149363	-0.040553239	-0.040190073
Nav1.5-S524Y	-0.111417836	-0.053240918	-0.051170053	-0.045932284	-0.041493325	-0.040550567	-0.04018815
100xCmax							
Nav1.5-WT	-0.152646353	-0.06877882	-0.065169988	-0.055782264	-0.047517528	-0.045721941	-0.04503116
Nav1.5-R1193Q	-0.152611897	-0.068748135	-0.065140805	-0.05575062	-0.047485081	-0.04569455	-0.045001478
Nav1.5-V1951L	-0.152607881	-0.06874559	-0.065137267	-0.055748223	-0.047481603	-0.045690555	-0.044996967
Nav1.5-S1103Y	-0.152607171	-0.068744327	-0.065134796	-0.055747126	-0.047481339	-0.045688586	-0.044998225
Nav1.5-S524Y	-0.152605532	-0.068743214	-0.06513385	-0.055746796	-0.047479383	-0.045686952	-0.044994861
