# Supplementary material for: Personalized in silico modeling of cardiac ion channel variants to predict drug-induced proarrhythmia risk
Source: Front Physiol. 2026 Aug 4;17:1828506. doi: 10.3389/fphys.2026.1828506 (PMC13495695; doi:10.3389/fphys.2026.1828506)
Supplement: Supplementary file 3 [file DataSheet3.pdf]

Table 10 - Heatmap-table displaying qNet values (µC/µF) for epicardial cells at different amiodarone concentrations. Red color shows the lowest qNet values, whereas green color shows the highest ones. Results in this table provide a comparison of all values relative to each other at one concentration.

qNet values for studied hERG and Nav1.5 alleles combinations							
0xCmax	hERG-WT	hERG-G648A	hERG-T623A	hERG-V659A	hERG-F656A	hERG-Y652A	hERG-S624A
Nav1.5-WT	0.058277008	0.058277008	0.058277008	0.058277008	0.058277008	0.058277008	0.058277008
Nav1.5-R1193Q	0.058277008	0.058277008	0.058277008	0.058277008	0.058277008	0.058277008	0.058277008
Nav1.5-V1951L	0.058277008	0.058277008	0.058277008	0.058277008	0.058277008	0.058277008	0.058277008
Nav1.5-S1103Y	0.058277008	0.058277008	0.058277008	0.058277008	0.058277008	0.058277008	0.058277008
Nav1.5-S524Y	0.058277008	0.058277008	0.058277008	0.058277008	0.058277008	0.058277008	0.058277008
1xCmax							
Nav1.5-WT	0.056379123	0.057943939	0.057981524	0.058083654	0.058163736	0.058183201	0.058189015
Nav1.5-R1193Q	0.056382723	0.057940703	0.057981893	0.058083174	0.058165423	0.058181808	0.058188455
Nav1.5-V1951L	0.056380848	0.057940501	0.057983212	0.058082837	0.058164577	0.05818406	0.058188698
Nav1.5-S1103Y	0.056386145	0.057940842	0.057982134	0.058085438	0.058166789	0.05818309	0.058187079
Nav1.5-S524Y	0.056382446	0.057941243	0.057982444	0.058082938	0.058166924	0.058183999	0.058187406
10xCmax							
Nav1.5-WT	0.040669846	0.054968719	0.055370197	0.056359345	0.057166928	0.057332576	0.057398727
Nav1.5-R1193Q	0.040673493	0.05497205	0.055374444	0.056362738	0.057169235	0.057337377	0.057401507
Nav1.5-V1951L	0.040672289	0.054971848	0.055372924	0.056362774	0.057170728	0.057336541	0.057403041
Nav1.5-S1103Y	0.040673953	0.054970153	0.055373977	0.056362402	0.057170296	0.05734035	0.057402916
Nav1.5-S524Y	0.040675427	0.054971895	0.055373679	0.056362247	0.057168494	0.057337905	0.057403885
20xCmax							
Nav1.5-WT	0.025743221	0.051757394	0.052538602	0.05447324	0.056067892	0.056403291	0.056531294
Nav1.5-R1193Q	0.025747612	0.051762857	0.052543059	0.054478038	0.056073319	0.056406323	0.056533511
Nav1.5-V1951L	0.025746925	0.051763897	0.052543577	0.054479102	0.056073584	0.056406434	0.056536188
Nav1.5-S1103Y	0.025747949	0.05176373	0.052543377	0.054477537	0.056072832	0.056406764	0.056533699
Nav1.5-S524Y	0.02574752	0.051764864	0.052542934	0.054479481	0.056071597	0.056407812	0.056535264
50xCmax							
Nav1.5-WT	-0.005622732	0.042662174	0.044466243	0.049003038	0.052833378	0.053646432	0.053956181
Nav1.5-R1193Q	-0.005610946	0.042675836	0.044477329	0.049017102	0.052845948	0.053656125	0.053968841
Nav1.5-V1951L	-0.005614764	0.042677717	0.044478252	0.049016955	0.052848968	0.053659989	0.053971043
Nav1.5-S1103Y	-0.005615697	0.042677909	0.044477931	0.049018025	0.05285033	0.053660867	0.05397058
Nav1.5-S524Y	-0.005611912	0.042679505	0.044479429	0.049019621	0.052849674	0.053659456	0.053971852
100xCmax							
Nav1.5-WT	-0.043992034	0.029136219	0.032278323	0.040465844	0.047649953	0.049201762	0.049799907
Nav1.5-R1193Q	-0.04397026	0.029161474	0.032305592	0.040494039	0.04767457	0.049226511	0.049823827
Nav1.5-V1951L	-0.043966893	0.029160387	0.032308183	0.04049781	0.047678672	0.049228297	0.049827504
Nav1.5-S1103Y	-0.043965442	0.029164701	0.032306928	0.040497965	0.047678503	0.049229705	0.049826786
Nav1.5-S524Y	-0.043962556	0.029165469	0.032308548	0.040498919	0.047679919	0.049232151	0.049828916

Table 11 - Heatmap-table displaying qNet values (μC/μF) for midwall cells at different amiodarone concentrations. Red color shows the lowest qNet values, whereas green color shows the highest ones. Results in this table provide a comparison of all values relative to each other at one concentration.

qNet values for studied hERG and Nav1.5 alleles combinations							
0xCmax	hERG-WT	hERG-G648A	hERG-T623A	hERG-V659A	hERG-F656A	hERG-Y652A	hERG-S624A
Nav1.5-WT	-0.129623395	-0.129623395	-0.129623395	-0.129623395	-0.129623395	-0.129623395	-0.129623395
Nav1.5-R1193Q	-0.129623395	-0.129623395	-0.129623395	-0.129623395	-0.129623395	-0.129623395	-0.129623395
Nav1.5-V1951L	-0.129623395	-0.129623395	-0.129623395	-0.129623395	-0.129623395	-0.129623395	-0.129623395
Nav1.5-S1103Y	-0.129623395	-0.129623395	-0.129623395	-0.129623395	-0.129623395	-0.129623395	-0.129623395
Nav1.5-S524Y	-0.129623395	-0.129623395	-0.129623395	-0.129623395	-0.129623395	-0.129623395	-0.129623395
1xCmax							
Nav1.5-WT	-0.132377061	-0.130109857	-0.130047643	-0.129904389	-0.129783626	-0.129759143	-0.129750551
Nav1.5-R1193Q	-0.132375863	-0.130107746	-0.130046695	-0.129903341	-0.129785451	-0.129760393	-0.129750945
Nav1.5-V1951L	-0.132375596	-0.130103046	-0.130045056	-0.129902385	-0.129782131	-0.129758074	-0.129748291
Nav1.5-S1103Y	-0.132376201	-0.130104734	-0.130045394	-0.12990226	-0.129782232	-0.12975978	-0.129749264
Nav1.5-S524Y	-0.13237645	-0.130108553	-0.130047944	-0.129902546	-0.129784232	-0.129758214	-0.129749625
10xCmax							
Nav1.5-WT	-0.156461627	-0.13444311	-0.133855282	-0.132406847	-0.131228529	-0.130982146	-0.130886197
Nav1.5-R1193Q	-0.15645909	-0.134441195	-0.133851557	-0.132403853	-0.131224935	-0.130980975	-0.130887263
Nav1.5-V1951L	-0.156459001	-0.134441715	-0.133851914	-0.132404011	-0.131224203	-0.130979281	-0.130886801
Nav1.5-S1103Y	-0.156457027	-0.134442341	-0.133852373	-0.132401945	-0.131223358	-0.130977951	-0.130886665
Nav1.5-S524Y	-0.15645828	-0.134437399	-0.133851626	-0.13240142	-0.131225368	-0.130979069	-0.130883282
20xCmax							
Nav1.5-WT	-0.181602684	-0.139216491	-0.138045214	-0.135170334	-0.132830009	-0.132342494	-0.132151511
Nav1.5-R1193Q	-0.181599495	-0.13921158	-0.138041755	-0.135168953	-0.132824066	-0.132334022	-0.132149158
Nav1.5-V1951L	-0.181599816	-0.139212692	-0.138041643	-0.135166588	-0.132828184	-0.132336787	-0.132148146
Nav1.5-S1103Y	-0.18159782	-0.139213032	-0.138041023	-0.135169574	-0.132825343	-0.132335905	-0.13214892
Nav1.5-S524Y	-0.181598066	-0.139212199	-0.138040104	-0.135167097	-0.13282719	-0.132335794	-0.13214772
50xCmax							
Nav1.5-WT	-0.245415638	-0.153261979	-0.150421513	-0.143379778	-0.13759316	-0.136387936	-0.135927962
Nav1.5-R1193Q	-0.245403882	-0.153251902	-0.150419846	-0.143369705	-0.137584929	-0.136379943	-0.135918662
Nav1.5-V1951L	-0.245399909	-0.15324991	-0.150412053	-0.143367838	-0.1375847	-0.136378898	-0.13592
Nav1.5-S1103Y	-0.24540557	-0.153251737	-0.150415403	-0.143369061	-0.137585564	-0.136378621	-0.135919333
Nav1.5-S524Y	-0.245401213	-0.1532489	-0.150409174	-0.143368842	-0.137582641	-0.13637784	-0.135916401
100xCmax							
Nav1.5-WT	-0.299418515	-0.175629508	-0.170260413	-0.156750906	-0.14544545	-0.14306172	-0.142151143
Nav1.5-R1193Q	-0.299390565	-0.175610688	-0.170241344	-0.156735272	-0.145422401	-0.143041358	-0.142132518
Nav1.5-V1951L	-0.299388514	-0.175608579	-0.170236629	-0.156732613	-0.145420681	-0.143041609	-0.142130501
Nav1.5-S1103Y	-0.299385608	-0.175603912	-0.170235102	-0.156730919	-0.145420668	-0.143041359	-0.142129527
Nav1.5-S524Y	-0.299383443	-0.175607536	-0.170233	-0.156730605	-0.145420047	-0.14303777	-0.142129514

Table 12. Summary of highest, lowest, and mean APD90 values across all allele combinations for all cell types

Cell Type	Concentration	Minimum	Maximum	Mean
**Endocardial**	**0xCmax**	272.53	272.53	272.53
	**1xCmax**	272.27	274.99	273.01
	**10xCmax**	273.22	294.15	277.42
	**20xCmax**	274.28	312.74	281.99
	**50xCmax**	277.36	357.99	293.76
	**100xCmax**	282.63	409.73	309.66

**Epicardial**	**0xCmax**	238.29	238.29	238.29
	**1xCmax**	238.24	240.7	238.79
	**10xCmax**	239.18	258.69	242.95
	**20xCmax**	240.11	276.9	247.33
	**50xCmax**	243.09	324.69	259.18
	**100xCmax**	247.76	383.8	275.68
**Midmyocardial**	**0xCmax**	331.69	331.69	331.69
	**1xCmax**	331.34	335.08	332.21
	**10xCmax**	332.98	364.89	339.03
	**20xCmax**	334.26	397.54	346.5
	**50xCmax**	338.91	483.07	367.13
	**100xCmax**	346.55	496.27	382.6
**Purkinje**	**0xCmax**	307.98	307.98	307.98
	**1xCmax**	307.76	311.16	308.7
	**10xCmax**	309.22	334.66	314.3
	**20xCmax**	310.41	358.57	320.01
	**50xCmax**	314.32	414.86	334.68
	**100xCmax**	320.62	478.76	354.51
**Atrial**	**0xCmax**	250.58	250.58	250.58
	**1xCmax**	250.1	251.71	250.75
	**10xCmax**	250.92	260.49	252.7
	**20xCmax**	251.2	267.95	254.6
	**50xCmax**	252.43	284.9	259.42
	**100xCmax**	255.39	300.25	265.51

Table 13. Summary of highest, lowest, and mean qNet values across all allele combinations for all endocardial, epicardial, and midmyocardial cells

Cell Type	Concentration	Minimum	Maximum	Mean
**Endocardial**	**0xCmax**	-0.03517	-0.03517	-0.03517
	**1xCmax**	-0.03738	-0.03527	-0.03567
	**10xCmax**	-0.05555	-0.03619	-0.03992
	**20xCmax**	-0.0727	-0.0372	-0.04417
	**50xCmax**	-0.11144	-0.04019	-0.05486
	**100xCmax**	-0.15265	-0.045	-0.06864
**Epicardial**	**0xCmax**	0.058277	0.058277	0.058277
	**1xCmax**	0.056379	0.058189	0.057847
	**10xCmax**	0.04067	0.057404	0.054184
	**20xCmax**	0.025743	0.056536	0.050506

	**50xCmax**	-0.00562	0.053972	0.041574
	**100xCmax**	-0.04399	0.049829	0.029242
**Midmyocardial**	**0xCmax**	-0.12962	-0.12962	-0.12962
	**1xCmax**	-0.13238	-0.12975	-0.13025
	**10xCmax**	-0.15646	-0.13088	-0.13575
	**20xCmax**	-0.1816	-0.13215	-0.14162
	**50xCmax**	-0.24542	-0.13592	-0.15748
	**100xCmax**	-0.29942	-0.14213	-0.17608
