# Supplementary material for: Personalized in silico modeling of cardiac ion channel variants to predict drug-induced proarrhythmia risk
Source: Front Physiol. 2026 Aug 4;17:1828506. doi: 10.3389/fphys.2026.1828506 (PMC13495695; doi:10.3389/fphys.2026.1828506)
Supplement: Supplementary file 4 [file DataSheet4.pdf]

Personalized In Silico Modeling of Cardiac Ion Channel Variants to Predict Drug-Induced Proarrhythmia Risk

Alia Henedi♦[1] & Jalal Cherkaoui♦[2], Stelian Camara Dit Pinto[3], Steven M. Levine[4], Mohammed Cherkaoui3, Nicolas R. Gallo*[3] & Kenza E. Benzeroual*[1]

Supplementary material

Table 14. Categorical regression Analysis

		Categorical regression analysis p-value results							
		APD90_Endo	APD90_Epi	APD90_Mid	APD90_Purk	APD90_Atri	qNet_Endo	qNet_Epi	qNet_Mid
R-squared:		0.689	0.666	0.717	0.689	0.731	0.709	0.708	0.692
P-value	Concentration	>0.001	>0.001	>0.001	>0.001	>0.001	>0.001	>0.001	>0.001
	hERG-WT	>0.001	>0.001	>0.001	>0.001	>0.001	>0.001	>0.001	>0.001
	hERG-G648A	0.646	0.553	0.818	0.871	0.675	0.743	0.76	0.636
	hERG-T623A	0.325	0.28	0.353	0.332	0.316	0.359	0.369	0.304
	hERG-V659A	0.019	0.025	0.011	0.013	0.02	0.017	0.017	0.017
	hERG-F656A	0.001	0.001	>0.001	>0.001	0.001	>0.001	>0.001	>0.001
	hERG-Y652A	>0.001	0.001	>0.001	>0.001	>0.001	>0.001	>0.001	>0.001
	hERG-S624A	>0.001	>0.001	>0.001	>0.001	>0.001	>0.001	>0.001	>0.001
	Nav1.5-WT	0.984	0.99	0.995	0.967	0.999	0.996	0.996	0.998
	Nav1.5-R1193Q	0.995	0.989	0.999	0.984	0.985	1	1	1
	Nav1.5-V1951L	0.995	0.995	0.996	0.982	0.993	0.999	0.999	1
	Nav1.5-S1103Y	0.99	0.989	0.986	0.966	0.997	0.999	0.999	1
	Nav1.5-S524Y	0.994	0.985	0.995	0.966	0.99	0.999	0.999	0.999
Coefficient	Concentration	13.1584	13.2876	18.3002	5.2759	16.4402	-0.0118	-0.0102	-0.0165
	hERG-WT	14.8581	15.3731	21.5871	5.6898	18.521	-0.013	-0.0112	-0.0192
	hERG-G648A	-0.4572	-0.6379	-0.3084	-0.0571	-0.52	0.0003	0.0002	0.0006
	hERG-T623A	-0.982	-1.1632	-1.2441	-0.3412	-1.2461	0.0008	0.0007	0.0013
	hERG-V659A	-2.3484	-2.4289	-3.4247	-0.8756	-2.9213	0.002	0.0017	0.003
	hERG-F656A	-3.4887	-3.5335	-5.2299	-1.4007	-4.3671	0.0031	0.0027	0.0045
	hERG-Y652A	-3.7341	-3.7663	-5.6155	-1.4837	-4.6868	0.0034	0.0029	0.0048

	hERG-S624A	-3.8476	-3.8433	-5.7645	-1.5315	-4.7797	0.0035	0.003	0.0049
	Nav1.5-WT	1.88E-02	-1.24E-02	8.20E-03	1.37E-02	1.80E-03	-3.91E-06	-3.21E-06	-2.64E-06
	Nav1.5-R1193Q	5.50E-03	1.43E-02	-1.20E-03	6.60E-03	-2.14E-02	4.23E-07	4.04E-07	2.02E-07
	Nav1.5-V1951L	-5.30E-03	-6.80E-03	6.60E-03	7.30E-03	1.02E-02	9.79E-07	7.77E-07	7.08E-07
	Nav1.5-S1103Y	-1.21E-02	-1.40E-02	-2.17E-02	-1.38E-02	-5.00E-03	1.14E-06	8.93E-07	7.28E-07
	Nav1.5-S524Y	-6.90E-03	1.89E-02	8.10E-03	-1.39E-02	1.44E-02	1.36E-06	1.14E-06	1.00E-06
		Step 1 refined regression							
R-squared:		0.689	0.665	0.717	0.689	0.731	0.709	0.708	0.692
P-value	Concentration	>0.001	>0.001	>0.001	>0.001	>0.001	>0.001	>0.001	>0.001
	hERG-WT	>0.001	>0.001	>0.001	>0.001	>0.001	>0.001	>0.001	>0.001
	hERG-V659A	0.211	0.277	0.13	0.209	0.141	0.17	0.167	0.206
	hERG-F656A	0.034	0.062	0.011	0.032	0.009	0.018	0.018	0.032
	hERG-Y652A	0.021	0.042	0.006	0.02	0.006	0.01	0.01	0.02
	hERG-S624A	0.017	0.037	0.005	0.017	0.004	0.008	0.008	0.017
Coefficient	Concentration	13.1584	13.2876	18.3002	16.4402	5.2759	-0.0118	-0.0102	-0.0165
	hERG-WT	15.5777	16.2736	22.3634	19.404	5.889	-0.0136	-0.0116	-0.0201
	hERG-V659A	-1.6288	-1.5284	-2.6484	-2.0383	-0.6765	0.0015	0.0013	0.0021
	hERG-F656A	-2.7691	-2.6329	-4.4537	-3.4841	-1.2015	0.0026	0.0023	0.0036
	hERG-Y652A	-3.0145	-2.8658	-4.8392	-3.8038	-1.2845	0.0028	0.0025	0.0039
	hERG-S624A	-3.128	-2.9428	-4.9882	-3.8967	-1.3323	0.0029	0.0025	0.004
		Step 2 refined regression							
R-squared:		0.686	0.663	0.713	0.689	0.727	0.709	0.708	0.692
P-value	Concentration	>0.001	>0.001	>0.001	>0.001	>0.001	>0.001	>0.001	>0.001
	hERG-WT	>0.001	>0.001	>0.001	>0.001	>0.001	>0.001	>0.001	>0.001
	hERG-F656A	0.071	0.11	0.032	0.068	0.025	0.043	0.043	0.068
	hERG-Y652A	0.045	0.076	0.017	0.042	0.015	0.025	0.025	0.044
	hERG-S624A	0.036	0.067	0.014	0.036	0.011	0.02	0.02	0.037
Coefficient	Concentration	13.1584	13.2876	18.3002	16.4402	5.2759	-0.0118	-0.0102	-0.0165
	hERG-WT	16.1206	16.7831	23.2462	20.0834	6.1144	-0.0141	-0.0121	-0.0208
	hERG-F656A	-2.2262	-2.1235	-3.5709	-2.8047	-0.976	0.0021	0.0018	0.0029
	hERG-Y652A	-2.4715	-2.3563	-3.9564	-3.1244	-1.059	0.0023	0.002	0.0032
	hERG-S624A	-2.5851	-2.4333	-4.1054	-3.2173	-1.1068	0.0024	0.0021	0.0033
