# Supplementary material for: Personalized in silico modeling of cardiac ion channel variants to predict drug-induced proarrhythmia risk
Source: Front Physiol. 2026 Aug 4;17:1828506. doi: 10.3389/fphys.2026.1828506 (PMC13495695; doi:10.3389/fphys.2026.1828506)
Supplement: Supplementary file 5 [file Table1.docx]

*Table 6 - Heatmap-table displaying APD90 values (ms) for midwall cells at various amiodarone concentrations. Red color shows the highest APD90 values, whereas green shows the lowest ones. Results in this table provide a comparison of all values relative to each other at one concentration.*

*Table 7 - Heatmap-table displaying APD90 values (ms) for Purkinje cells at various amiodarone concentrations. Red color shows the highest APD90 values, whereas green shows the lowest ones. Results in this table provide a comparison of all values relative to each other at one concentration.*
